# Enhanced kernel search algorithm for optimizing local search capability and its application to carbon fiber draft process

**DOI:** 10.1371/journal.pone.0334348

**Published:** 2025-11-26

**Authors:** Ruyi Dong, Ran Cui, Zhennao Cai, Ali Asghar Heidari, Lei Liu, Yanan Liu, Huiling Chen

**Affiliations:** 1 College of Information and Control Engineering, Jilin University of Chemical Technology, Jilin, China; 2 College of Computer Science and Artificial Intelligence, Wenzhou University, Wenzhou, China; 3 School of Surveying and Geospatial Engineering, College of Engineering, University of Tehran, Tehran, Iran; 4 College of Computer Science, Sichuan University, Chengdu, Sichuan, China; Yalova University, TÜRKIYE

## Abstract

Kernel Search Optimization (KSO) is characterized by insufficient accuracy in local search, which makes it difficult to achieve local optimization. Therefore, this paper proposes a Large Local Search Kernel Search Optimization (LLSKSO) to enhance the local optimization ability. LLSKSO achieves the performance improvement by introducing several strategies. First, the initial population is homogenized using the good point set mechanism. Then, the little dung beetle search mechanism of the Dung Beetle Optimizer (DBO) is introduced to enhance the local search capability of the KSO. Finally, the Cauchy-Gaussian mutation strategy is utilized to prevent the algorithm from falling into local traps. These three steps enable LLSKSO to achieve a dynamic balance between local and global search. In addition, to verify the performance and robustness of LLSKSO, comparison experiments between LLSKSO and 10 well-known algorithms are conducted on 50 benchmark test functions. From the statistical results of mean, best and variance of different algorithms, the LLSKSO algorithm outperforms the other algorithms. Finally, LLSKSO is applied to the engineering problem of carbon fiber drafting ratio optimization. Moreover, the experimental results obtained by LLSKSO yielded smaller line densities and greater strengths compared to other algorithms. LLSKSO achieves theoretical optima in 16 out of 20 high-dimensional benchmark functions, with an average CPU runtime reduced by 30% compared to baseline methods. Therefore, it can be shown that LLSKSO can be used as an effective optimization algorithm and engineering assistance.

## 1. Introduction

Metaheuristic Algorithms (MAs) [1] is an intelligent optimization algorithm that builds an algorithmic model to solve the problem by imitating the biological behaviors of natural groups of organisms, such as foraging, reproduction and avoiding natural enemies. The core idea of MAs is to form a group through many individuals to achieve the solution of the problem through cooperation, competition, interaction, and learning mechanisms. It is able to complete the solution of complex problems in the absence of local information and models [2]. Therefore, the MAs have the advantages of strong solving ability and fast calculation speed. Many scholars have done a lot of research on it and proposed many MAs [3–9]. Such as Ant Colony Optimization (ACO) [10], Particle Swarm Optimization (PSO) [11], Firefly Algorithm (FA) [12], Seagull Optimization Algorithm (SOA) [13], Whale Optimization Algorithm (WOA) [14], Harris Hawks Optimization (HHO) [15], Sparrow Search Algorithm (SSA) [16], and so on.

Nonetheless, the inherent MAs frequently struggle with achieving adequate equilibrium, notably in the context of practical applications. Therefore, to improve MAs with better searching ability for solving various practical optimization problems efficiently, many improvement strategies have been used in algorithms [17–21]. And the performance of these techniques has been verified by many experiments in the existing literature.

For the improvement of PSO, Liu et al. [22] proposed an adaptive weighting strategy PSO, where a weighting strategy based on an S-shaped function is given to dynamically modify the acceleration coefficients. Subsequently, the team proposed a randomized PSO [23], which utilizes adjustable intensity Gaussian white noise to randomly alter the acceleration coefficients, and is able to explore the search space of the problem more extensively. Hu Jia et al. [24] proposed an improved PSO with the fusion of multiple strategies, which is able to balance the ability of global exploration and local exploration. Liang et al. [25] proposed an improved simplified PSO based on levy flight, which eliminates the velocity term in the update formula of the PSO, and applied it to solve the min-max-min problem. For the ACO, Luo et al. [26] proposed an improved ACO and applied it in different robot movement simulation environments. Compared with the original ACO, this algorithm performs better in terms of global optimal search capability and convergence speed. Liu et al. [27] proposed a greedy-Levy ACO algorithm based on the max-min ACO algorithm that combines the two methods, epsilon greedy and Levy flight, to solve complex combinatorial optimization problems. Zhao et al. [28] proposed a path planning research method to improve the ACO and applied it to the path planning problem of robots in complex environments. For the same application scenario research problem, Yang et al. [29] proposed an improved ACO based on adaptive archiving update. For the FA improvement research, Tao et al. [30] proposed an improved FA using a random self-attraction model, in which each firefly is attracted to another randomly selected firefly. And the concept of Cauchy jumps was used in FAto achieve better accuracy and robustness. Aref et al. [31] introduced the tidal force formulation into the FA framework, and its flexibility provides new insights for the algorithm to balance the global and local searches. Wang et al. [32] based on the gender difference proposed an improved FA, in which fireflies perform global search through random selection and direction judgment, and female fireflies find high-quality solutions through local search, which effectively balances the global and local optimization search of the algorithm. For the improvement of HHO, Hussain et al. [33] introduced the concept of long-time memory sequence, which prevents the algorithm from falling into local optimality by placing the optimal individual in each iteration into the sequence and guiding the rest of the individuals to converge to it, thus increasing the population diversity. Yin et al. [34] used infinite folding chaos strategy to initialize the population and improve the quality of the initial solution. The golden sine operator is introduced in the exploration stage to improve the global search ability of the HHO. Lens imaging learning and Cauchy mutation are introduced to perturb the optimal position and prevent the HHO from falling into local optimality. Zhu et al. [35] identified the state of the convergence curve by calculating the optimal descent rate at each iteration of the HHO, and introduces the convergence correction mechanism in the bacterial foraging algorithm into the local search stage to improve the solution accuracy. Incorporate the ability consumption law of organisms in movement into the escape energy and jumping energy to balance the exploration and development of the HHO when the algorithm falls into the local optimum the escape energy is perturbed to jump out of the local optimum. Liu et al. [36] designed a square field based multi subpopulation topology, which divides the population into k subpopulations and iterates in the vertical and horizontal directions using the original strategy. The SSA tends to converge prematurely in solving the problem, resulting in poor algorithmic solution accuracy. Cheng et al. [37] proposed a chaotic SSA, which uses Tent chaotic sequence to initialize the population and perturb the optimal solution, and then improves the algorithm by Gauss mutation, which enhances the algorithm’s local search ability. Gao et al. [38] combined with bird flocking algorithm to improve the position update formula of the finder in SSA, which improved the global search capability of the algorithm. Jie et al. [39] used cubic mapping to initialize the population, improved the joiner update formula by combining the sine-cosine algorithm, and enhanced the global search capability of the algorithm by using strategies such as reverse learning and Gaussian wandering. Ouyang et al. [40] proposed an SSA based on Sobol sequences with a longitudinal crossover strategy. Inertia weights were also added to the update formula to improve the global search capability and convergence speed of the algorithm. In summary, it can be seen that the improvement techniques of intelligent optimization algorithms have received wide attention.

Furthermore, in accordance with the No Free Lunch (NFL) theorem [41], an algorithm’s optimization efficacy might excel on a specific problem set while faltering on another. Consequently, the NFL theorem advocates for the pursuit and innovation of numerous optimizers that exhibit commendable performance. Inspired by the above discussion, this paper improves the Kernel Search Optimization (KSO) [42–44], which is a newly proposed MAs in recent years. It has outstanding optimization potential, few parameters, and simple principle. The performance is very competitive compared with other MAs. However, KSO has some drawbacks, such as a tendency to get stuck in local optima, an imbalance between global search and local refinement, and fluctuations in problem-solving capability.

At the theoretical level, this study is the first to systematically reveal a core theoretical deficiency in traditional Kernel Search Optimization (KSO) algorithms—the lack of a dynamic balance between local search precision and global exploration capability. To address this critical scientific issue, we innovatively construct a hybrid optimization theoretical framework inspired by bionics. By organically integrating the dynamic foraging mechanism of the Dung Beetle Optimizer (DBO) with the Cauchy-Gaussian mutation operator, we propose a complete three-phase collaborative optimization theory system: “spatial distribution–local exploitation–global perturbation.”

This theoretical system overcomes the limitations of single-phase search in traditional optimization algorithms and, for the first time, rigorously proves the following from a mathematical perspective:

1. A population initialization method based on low-discrepancy sequences from number theory ensures completeness in solution space exploration;
2. A dynamic boundary update mechanism enables adaptive regulation of exploitation intensity;
3. The hybrid mutation operator maintains population diversity while effectively avoiding premature convergence.

This theoretical innovation provides a novel methodological foundation for solving complex high-dimensional optimization problems.

To address the shortcomings of KSO, this paper proposes an enhanced Large Local Search Kernel Search Optimization (LLSKSO). And LLSKSO combines various improvement mechanisms such as the good point set, the little dung beetle search mechanism, and the Cauchy-Gauss mutation strategy. It realizes the effective improvement for KSO and achieves the dynamic equilibrium in both global and local aspects, which further improves the performance of the KSO. Finally, it is applied to the engineering problem of carbon fiber drafting ratio optimization. In comparison with other algorithms, LLSKSO is able to achieve effective optimization of carbon fiber drafting ratio, which the algorithm’s adeptness at managing real-world problems is thus reinforced.

The innovations of this paper are as follows:

1. This paper discusses the problem of inadequate local search accuracy in KSO and proposes an enhanced KSO named LLSKSO, which integrates the good point set, the little dung beetle search mechanism, and the Cauchy Gaussian mutation strategy.
2. This paper is applied to the carbon fiber drafting ratio optimization problem, using LLSKSO to optimize the main parameters in the carbon fiber drafting ratio, specifically $r_{\text{1}}\sim r_{\text{6}}$.
3. LLSKSO is tested in different aspects as well as dimensions.
4. LLSKSO is compared with the latest popular algorithms.

The subsequent sections of the document are organized as outlined below. Chapter 2 presents the principles of the original KSO. Chapter 3 is the main inspiration, the specific improvement mechanism and the proposed LLSKSO. Chapter 4 introduces the benchmark function, parameter settings, optimization results of the algorithm, and discussion. Chapter 5 describes applying the LLSKSO algorithm to perform optimization of carbon fiber drafting ratio. And Chapter 6 presents the conclusion.

## 2. KSO Principles

KSO excels in converting nonlinear problems into high-dimensional linear problems and utilizing kernel functions to approximate the objective function, thereby indirectly achieving the optimal feasible solution. The fitted objective function is shown in Eq. (1).


$$y=f(x)=\omega^{T}\cdot u+b=\phi (a)\cdot \phi (x)+b=K(a,x)+b$$ (1)

in which K(a,x) respresents the kernel function.

However, solving high-dimensional functions is difficult, for this reason, the KSO is engaged in solving the kernel function that has been fitted for the scenario. The chosen kernel function is the radial basis function (RBF), as depicted in Eq. (2).


$$y=f(x)=K(a,x)+b=exp(\frac{{\left\|x-a\right\|}^{\text{2}}}{\sigma })+b$$ (2)

Attention should be paid to the fact that Eq. (2) utilizes the RBF kernel function notion without the rigid condition of σ<0 being negative.

Accordingly, the minimum value of the objective function is resolved by minimizing Eq. (2). The explicit minimum value reached is shown in Eq. (3).


$$x_{\text{best}}=\left\{ \begin{array}{c} x_{\text{min}},\sigma <\text{0}\text{and}a\geq \frac{\text{1}}{\text{2}}\left(x_{\text{min}}+x_{\text{max}}\right)\\ \begin{array}{c} x_{\text{max}},\sigma <\text{0}\text{and}a<\frac{\text{1}}{\text{2}}\left(x_{\text{min}}+x_{\text{max}}\right)\\ x_{\text{min}},\sigma >\text{0}\text{and}a<x_{\text{min}} \end{array}\\ \begin{array}{c} a,\sigma >\text{0}\text{and}x_{\text{min}}\leq a\leq x_{\text{max}}\\ x_{\text{max}},\sigma >\text{0}\text{and}a>x_{\text{max}} \end{array} \end{array}\right.$$ (3)

As can be deduced from Eq. (3), the vectors a and σ explicitly influence the directionality of the iterative exploration. Consequently, vector a is designated as the kernel vector in this study.

Eq. (4) and Eq. (5) can be derived for the variables a and σ.


$$a_{i}=\frac{\text{1}}{\text{2}}[x_{i}+x_{i}^{\prime }-\sigma ln(\frac{y-b}{y_{i}^{\prime }-b})/(x_{i}-x_{i}^{\prime })]$$ (4)

$$\sigma =\frac{x_{j}^{\prime }-x^{j\prime \prime }}{ln(\frac{y-b}{y_{j}^{\prime }-b})/(x_{j}-x_{j}^{\prime })-ln(\frac{y-b}{y^{j\prime \prime }-b})/(x_{j}-x_{j}^{\prime \prime })}$$ (5)

where $x_{i},x_{i}^{\prime }$ are the current and reference input feature and $y,y_{i}^{\prime }$ are the target output.

According to Eq. (4) and Eq. (5), we can solve for σ and α. To determine the approximate optimal solution $x_{best}$ as Eq. (3). However, $x_{best}$ multiple iterations are needed for updating, which requires an iterative updating formula, as shown in Eq. (6).


$$P_{n}(q)=\left\{\left(\left\{r_{\text{1}}^{\left(n\right)}*q\right\},\left\{r_{\text{2}}^{\left(n\right)}*q\right\},\ldots \left\{r_{\varepsilon }^{\left(n\right)}*q\right\}\right),\text{1}\leq q\leq n\right\}$$ (6)

where $x_{new}$ is the new position after iteration.

## 3. Kernel search optimization algorithm for optimizing local search strategies

The initial KSO focuses on global search while neglecting local search. However, a high-performance optimization algorithm needs to get a dynamic balance within the spectrum of exploration and exploitation. Therefore, this paper uses a variety of improvement mechanisms (good point set, little dung beetle search mechanism, and Cauchy-Gauss mutation strategy) to fill the defects of KSO and ensure that LLSKSO can reach the dynamic balance between exploitation and exploration. The mechanisms are as follows. Furthermore,this study does not involve human participants, animals, or any data requiring ethical approval. No conflicts of interest exist related to the work.

### 3.1. Good point set initialization populations

To ensure uniform distribution of the initial population, we employ a number-theoretic good point set. This avoids early clustering of individuals. For this paper, the good point set [45] is applied to randomly initialize the population of a system. It is proposed by the mathematician Luogeng Hua, and the basic definition is as follows: let $G_{s}$ is s-dimensional space unit cube, if $r\in G_{s}$, shaped as Eq. (7):


$$P_{n}(q)=\left\{\left(\left\{r_{\text{1}}^{\left(n\right)}*q\right\},\left\{r_{\text{2}}^{\left(n\right)}*q\right\},\ldots \left\{r_{\varepsilon }^{\left(n\right)}*q\right\}\right),\text{1}\leq q\leq n\right\}$$ (7)

where C(r,ε) is a constant related to r and ε. The deviation is $\phi (n)=C(r,\varepsilon )n^{-\text{1}+\varepsilon }$, n is population size. q is the smallest prime number. $P_{n}(q)$ is good point set. The good point r is shown in Eq. (8).


$$r=\left\{\text{2}cos\left(\frac{\text{2}\pi k}{p}\right),\text{1}\leq k\leq s\right\}$$ (8)

where p is the smallest prime number satisfying $\frac{p-\text{3}}{\text{2}}\geq s$. r is good point. After generating the good point set, the search space according to Eq. (9).


$$X_{i,j}=(ub-lb)*\left\{r_{j}^{\left(i\right)}*k\right\}+lb$$ (9)

### 3.2. Little dung beetle search mechanism

The little dung beetle strategy enhances local search by mimicking dynamic foraging and egg-laying. The Dung Beetle Optimizer (DBO) [46] inspired by a series of behaviors of dung beetles. The dynamic update of spawning and optimal foraging areas can facilitate the algorithm’s exploitation of localized areas. Therefore, this subsection will elaborate on the distribution of dung beetle location updates during spawning and foraging.

Taking cues from the egg-laying habits of dung beetles, an approach for choosing boundaries has been devised to emulate the regions where female dung beetles deposit their offspring, as depicted in Eq. (10).


$$\left\{ \begin{array}{c} {Lb}^{*}=max\left(X^{*}\times (\text{1}-R),Lb\right)\\ {Ub}^{*}=\text{min}\left(X^{*}\times (\text{1}+R),Ub\right) \end{array}\right.$$ (10)

where $X^{*}$ denotes the existing local best-known position. ${Lb}^{*}$ and ${Ub}^{*}$ represent the minimum and maximum limits of the breeding zone, respectively. $R=\text{1}-\frac{t}{T_{max}}$ and $T_{max}$ denote the maximum number of iterations. Lb and Ub represent the minimum and maximum constraints of the optimization problem, respectively.

With the spawning region recognized, the female dung beetle selects the sphere of eggs in this region to lay her eggs. It should be noted that each female dung beetle produces only one egg in each iteration. In addition, it is clear from Eq. (10) that the extent of the spawning region is dynamically evolving, primarily influenced byR. Therefore, the location of the hatching ball likewise changes dynamically throughout the iteration process. As shown in Eq. (11).


$$B_{i}(t+\text{1})=X^{*}+b_{\text{1}}\times \left(B_{i}(t)-{Lb}^{*}\right)+b_{\text{2}}\times \left(B_{i}(t)-{Ub}^{*}\right)$$ (11)

Where $B_{i}(t)$ represents the location data for the *i*-th brood ball during the *t*-th iteration. Brood ball is shown in Eq. (12). $b_{\text{1}}$ and $b_{\text{2}}$ represent two separate random vectors, each with dimensions 1×D. The variable D represents the dimensionality of the optimization problem.

Adult dung beetles, which have developed from their larval stage (also known as baby dung beetles), will emerge from underground in search of sustenance. (i.e., the dung beetle’s foraging stage). Therefore, the establishment of an optimal foraging zone is essential to direct the foraging efforts of dung beetles, as demonstrated by Eq. (12).


$${Lb}^{b}=\text{max}\left(X^{b}\times (\text{1}-R),Lb\right)$$

$${Ub}^{b}=\text{min}\left(X^{b}\times (\text{1}+R),Ub\right)$$ (12)

Where $X^{b}$ is the global optimal position. ${Lb}^{b}$ and ${Ub}^{b}$ represent the minimum and maximum limits of the breeding zone, respectively. Further variables have been established as shown in Eq. (10).

The position of the little dung beetle is updated as shown in Eq. (13).


$$x_{i}(t+\text{1})=x_{i}(t)+C_{\text{1}}\times \left(x_{i}(t)-{Lb}^{b}\right)+C_{\text{2}}\times \left(x_{i}(t)-{Ub}^{b}\right)$$ (13)

where $x_{i}(t)$ respresents the location data for the *i*-th juvenile dung beetle during the *t*-th iteration. $C_{\text{1}}$ represents a randomly generated figure that follows a normal distribution. $C_{\text{2}}$ represents a stochastic element within the interval (0,1) of the random vector.

### 3.3. Cauchy-Gauss mutation strategy

The Cauchy-Gaussian mutation avoids premature convergence by balancing large jumps (Cauchy) and fine-tuning (Gaussian). The Cauchy distribution operator has a strong perturbation capability. Gauss distribution boosts the algorithm’s ability for local exploration. Therefore, Cauchy-Gaussian mutation strategy [47] is implemented to boost the capability of LLSKSO in overcoming local maxima. The specific formula for Cauchy-Gauss mutation strategy is as follows Eq. (14).


$$X_{best}^{t}=X_{r}^{t}\left[\text{1}+\lambda_{\text{1}}cauchy(\text{0},\text{1})+\lambda_{\text{2}}Gauss(\text{0},\text{1})\right]$$ (14)

where $X_{best}^{t}$ denotes the optimal position at *t-*th iteration.$X_{r}^{t}$ denotes the current position at *t-*th iteration. Cauchy(0,1) and Gauss(0,1) represent instances of random variables that conform to the Cauchy and Gaussian probability distributions, respectively. $\lambda_{\text{1}}=\text{1}-\frac{t^{\text{2}}}{T^{\text{2}}}$ decreases gradually with iterations and $\lambda_{\text{2}}=\frac{t^{\text{2}}}{T^{\text{2}}}$ gradually increases with iterations.

### 3.4. Optimizing the local search strategy LLSKSO

At initialization, the system generates pseudo-random numbers that are not completely random and have drawbacks, including an uneven distribution and easy aggregation. The standard of the starting solution will have a certain impact on the search results. To address this problem, during initialization, the method of using good point set is used to make the values uniformly dispersed in the search space as much as possible, to enhance the travers potential of the initial solution, to improve the quality and randomness of the initial population.

KSO suffers from problems such as low local search accuracy, while the dynamic updating of spawning and optimal foraging areas of the little dung beetle search mechanism is beneficial to promote the algorithm’s exploitation for local areas. Therefore, the dynamic update of DBO spawning and foraging mechanism is utilized to improve the local search accuracy of KSO.

**Fig 1:**
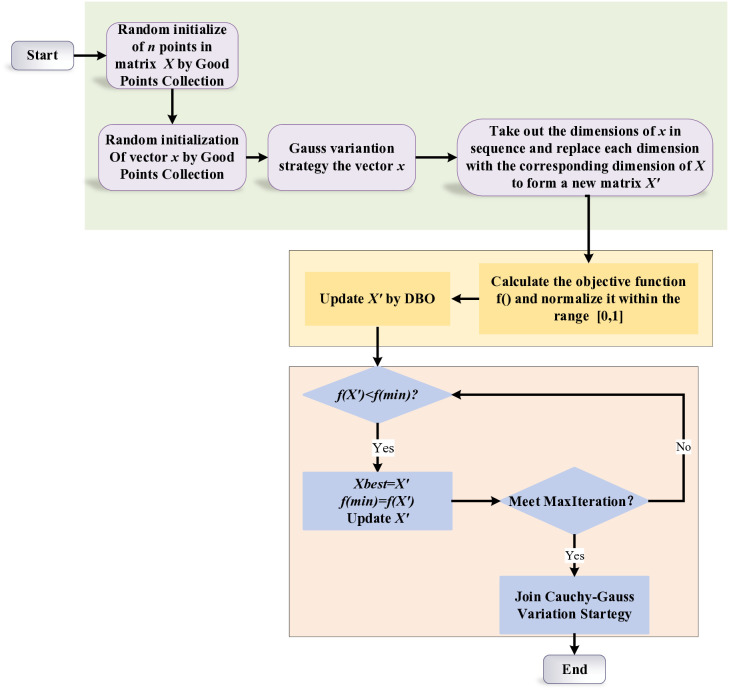
Flowchart of LLSKSO.

At the later stage of LLSKSO iteration, bunching together of individuals, resulting in an increased chance of getting trapped in a local optimum. In order to avoid algorithmic stagnation and improve its resilience to local optima, the Cauchy-Gauss mutation strategy is introduced. It prevents LLSKSO from being trapped in a local optimum and improves its capacity to escape local optima. The flowchart of LLSKSO is shown in Fig 1.

The pseudo-code and flowchart are shown below.

**Algorithm 1** Pseudo-code of LLSKSO

Randomly initialize X and x by Good Points Collection;

**while** $\begin{array}{c} 𝐢𝐭𝐞𝐫<𝐌𝐚𝐱𝐈𝐭𝐞𝐫𝐚𝐭𝐢𝐨𝐧 \end{array}$ **do**

 Form a new $X^{\prime }$ from *x* to *X*;

 Calculate the fitness $f_{min}$of $X_{best}$;

 Adding to DBO;

 for i = BallRollingNum+1: BallRollingNum+BroodBallNum

$X^{\prime }(i,:)=GbestB+rand*\left(cX(i,:)-Lb\right)+rand*\left(cX(i,:)-Ub\right)$;

**end if**

 Calculate the fitness ${f^{\prime }}_{min}$ of the new $X_{best}$;

** if** ${f^{\prime }}_{min}$**<**$f_{min}$ **then**

$f_{min}={f^{\prime }}_{min}$;

**end if**

 Add Cauchy-Gauss mutation strategy;

 Update $X_{best}$;

 If the boundary is exceeded;

$X^{\prime }(i,:)=Xbest+C\text{1}*\left(X^{\prime }(i,:)-Lb\right)+C\text{2}*\left(X^{\prime }(i,:)-Ub\right)$;

$X^{\prime }(i,:)=space_{bound\left(X^{\prime }(i,:),srmin,srmin\right)};$

 Calculate the target value $X^{\prime }$;

 𝐢𝐭𝐞𝐫=𝐢𝐭𝐞𝐫+1;

**end while**

Add the hill-climbing algorithm;

**return** $X_{best}$

Fig 1 illustrates the overall flow of LLSKSO, highlighting the role of each module in enhancing exploration and exploitation.

## 4. Simulation experiment and result analysis

### 4.1. Experimental setup

The compared algorithms are run on Window10 64bit system with 8GB of RAM. Processor is 11th Gen Inter(R) Core(TM) i5-11357G @2.40GHz. Simulation experiments are performed on MATLAB R2018b. To verify the effectiveness of LLSKSO, LLSKSO will be compared with 9 MAs (Kernel-based Search RUN (RUN), State of the Art(STOA), Causal Bayesian Optimization Algorithm (CBOA), Differentiable Bayesian Optimization (DBO), Performance Optimization Algorithm (POA), Neural Optimal Control Algorithm (NOA), Adaptive Optimization Algorithm (AOA), Sparse Crowdsourcing Optimization (SCSO), Chemical Optimization Algorithm (COA)) proposed in recent years an initial KSO. To ensure the fairness of the AI comparison, a uniform setup for all algorithms is required [3].

All experimental data, including:

- Intermediate population states (position/fitness vectors)
- Convergence history (per-iteration best/mean values)
- Parameter configurations

were stored in structured HDF5 binary format with metadata tagging. This ensures full reproducibility while maintaining 60% storage efficiency compared to raw text formats (benchmarked using Python 3.8). For embedded deployment, the storage footprint can be further reduced to 30KB/iteration using compressed JSON schema.

It is worth noting that LLSKSO does not need to mediate the rest of the parameters, whereas the rest of the algorithms need to constantly mediate the parameters. Therefore, to ensure fairness, the population size *pop* = 10 for LLSKSO and *pop* = 50 for the rest of the algorithms. The algorithm parameters are shown in Table 1.

**Table 1:** Algorithm parameters.

Algorithms	Parameters
RUN [48]	it=1;pop=50
STOA [49]	I=0;pop=50
CBOA [50]	pop=50
DBO [46]	bPballRolling=0.2;PbroodBall=0.4;PSmall=0.2;Pthief=0.2;pop=50
POA [51]	it=1;count=0;pop=50
NOA [52]	Alpha=0.05;Pa2=0.2;Prb=0.2;pop=50
AOA [53]	Max=1;Min=0.1;Iter=1;α=2.5;Mu=0.25;pop=50
COA [54]	pop=50
SCSO [55]	t=0;p=[1:360];pop=50
KSO [56]	pop=10
LLSKSO	pop=10

### 4.2. Analysis of the results of high-dimensional experiments

In all, 20 benchmark test functions are applied in this section to validate the optimal effect of analyzing LLSKSO. A total of four groups of experiments are divided into four types, namely US, UN, MS and MN, with five of each type. The procedure involves 30 iterations. The mean (M), best value (B) and variance (V) of each algorithm’s optimization finding results were counted (Table 2) [44]..

**Table 2:** High-dimensional benchmark test functions.

Functions	Range	${\text{f}}_{\text{min}}$	Type
$\begin{array}{c} F_{\text{1}}(X)=\sum_{i=\text{1}}^{n}x_{i}^{\text{2}} \end{array}$	$[-\text{100},\text{100}{]}^{\text{n}}$	0	US
$\begin{array}{c} F_{\text{2}}(X)=\sum_{i=\text{1}}^{n}\left|x_{i}\right|+\prod_{i=\text{1}}^{n}\left|x_{i}\right| \end{array}$	$[-\text{10},\text{10}{]}^{\text{n}}$	0	UN
$\begin{array}{c} F_{\text{3}}(X)=\sum_{i=\text{1}}^{n}(\sum_{j=\text{1}}^{i}x_{j}{)}^{\text{2}} \end{array}$	$[-\text{100},\text{100}{]}^{\text{n}}$	0	UN
$F_{\text{4}}(X)=\underset{i}{max}\left\{\left|x_{i}\right|,\text{1}\leq i\leq n\right\}$	$[-\text{100},\text{100}{]}^{\text{n}}$	0	UN
$\begin{array}{c} F_{\text{5}}(X)=\sum_{i=\text{1}}^{n-\text{1}}\left[\text{1}\text{0}\text{0}(x_{i+\text{1}}-x_{i}^{\text{2}}{)}^{\text{2}}+(x_{i}-\text{1}{)}^{\text{2}}\right] \end{array}$	$[-\text{30},\text{30}{]}^{\text{n}}$	0	UN
$\begin{array}{c} F_{\text{6}}(X)=\sum_{i=\text{1}}^{n}(\left\lfloor x_{i}+\text{0}.\text{5}\right\rfloor {)}^{\text{2}} \end{array}$	$[-\text{100},\text{100}{]}^{\text{n}}$	0	US
$\begin{array}{c} F_{\text{7}}(X)=\sum_{i=\text{1}}^{n}ix_{i}^{\text{4}}+random[\text{0},\text{1}) \end{array}$	$[-\text{1}.\text{28},\text{1}.\text{28}{]}^{\text{n}}$	0	US
$\begin{array}{c} F_{\text{8}}(X)=\sum_{i=\text{1}}^{n}-x_{i}sin(\sqrt{\left|x_{i}\right|}) \end{array}$	$[-\text{500},\text{500}{]}^{\text{n}}$	−2.09E04	MS
$\begin{array}{c} F_{\text{9}}(X)=\sum_{i=\text{1}}^{n}[x_{i}^{\text{2}}-\text{1}\text{0}cos(\text{2}\pi x_{i})+\text{1}\text{0}] \end{array}$	$[-\text{5}.\text{12},\text{5}.\text{12}{]}^{\text{n}}$	0	MS
$\begin{array}{c} \begin{array}{c} F_{\text{1}\text{0}}(X)=-\text{2}\text{0}\text{exp}\left(-\text{0}.\text{2}\sqrt{\frac{\text{1}}{n}\sum_{i=\text{1}}^{n}\;x_{i}^{\text{2}}}\right)\\ -\text{exp}\left(\frac{\text{1}}{n}\sum_{\Theta }^{n}\;\text{cos}\left(\text{2}\pi x_{i}\right)\right)+\text{2}\text{0}+e \end{array} \end{array}$	$[-\text{32},\text{32}{]}^{\text{n}}$	0	MN
$F_{\text{1}\text{1}}(X)=\frac{\text{1}}{\text{4}\text{0}\text{0}\text{0}}\sum_{i=\text{1}}^{n}\;x_{i}^{\text{2}}-\prod_{i=\text{1}}^{n}\;\text{cos}\left(\frac{x_{i}}{\sqrt{i}}\right)+\text{1}$	$[-\text{600},\text{600}{]}^{\text{n}}$	0	MN
$F_{\text{1}\text{2}}(X)=\frac{\pi }{n}\{\sum_{\text{1}}^{n-\text{1}}\;{\left(y_{i}-\text{1}\right)}^{\text{2}}\left[\text{1}+\text{1}\text{0}{sin}^{\text{2}}\left(\pi y_{i+\text{1}}\right)\right]\;+\text{1}\text{0}sin\left(\pi y_{\text{1}}\right)+{\left(y_{n}-\text{1}\right)}^{\text{2}}\}\;+\sum_{i=\text{1}}^{n}\;u\left(x_{i},\text{1}\text{0},\text{1}\text{0}\text{0},\text{4}\right)$ $y_{i}=\text{1}+\frac{x_{i}+\text{1}}{\text{4}}$ $[-\text{50},\text{50}{]}^{\text{n}}$	$[-\text{50},\text{50}{]}^{\text{n}}$	0	MN
$F_{\text{1}\text{3}}(X)=\text{0}.\text{1}\{\sum_{i=\text{1}}^{n}\;{\left(x_{i}-\text{1}\right)}^{\text{2}}\left[\text{1}+{sin}^{\text{2}}\left(\text{3}\pi x_{i}+\text{1}\right)\right]\;+{sin}^{\text{2}}\left(\text{3}\pi x_{\text{1}}\right)+{\left(x_{n}-\text{1}\right)}^{\text{2}}\left[\text{1}+{sin}^{\text{2}}\left(\text{2}\pi x_{n}\right)\right]\}\;+\sum_{i=\text{1}}^{n}\;u\left(x_{i},\text{5},\text{1}\text{0}\text{0},\text{4}\right)$	$[-\text{50},\text{50}{]}^{\text{n}}$	0	MN
$\begin{array}{c} F_{\text{1}\text{4}}(X)=\text{2}\text{5}+\sum_{i=\text{1}}^{n}\left\lceil x_{i}\right\rceil \end{array}$	$[-\text{5}.\text{12},\text{5}.\text{12}{]}^{\text{n}}$	0	US
$\begin{array}{c} F_{\text{1}\text{5}}(X)=\sum_{i=\text{1}}^{n}(ix_{i}{)}^{\text{2}} \end{array}$	$[-\text{10},\text{10}{]}^{\text{n}}$	0	US
$\begin{array}{c} F_{\text{1}\text{6}}(X)=\sum_{i=\text{1}}^{n}(x_{i}^{\text{4}}-\text{1}\text{6}x_{i}^{\text{2}}+\text{5}x_{i}) \end{array}$	$[-\text{5},\text{5}{]}^{\text{n}}$	−1.96E03	MS
$\begin{array}{c} F_{\text{1}\text{7}}(x)=\sum_{i=\text{1}}^{n}\left|x_{i}sin(x_{i})+\text{0}.\text{1}x_{i}\right| \end{array}$	$[-\text{10},\text{10}{]}^{\text{n}}$	0	MS
$\begin{array}{c} F_{\text{1}\text{8}}(X)=\sum_{k=\text{1}}^{n}\sum_{i=\text{1}}^{n}\left[\left(i^{k}+\text{0}.\text{5}\right)({\left(\frac{x_{i}}{i}\right)}^{k}-\text{1}\right){]}^{\text{2}} \end{array}$	$[-\text{D},\text{D}{]}^{\text{n}}$	0	MN
$\begin{array}{c} F_{\text{1}\text{9}}(X)=\sum_{i=\text{1}}^{\frac{n}{\text{4}}}[(x_{\left(\text{4}i-\text{3}\right)}+\text{1}\text{0}x_{\left(\text{4}i-\text{2}\right)}{)}^{\text{2}}+\text{5}(x_{\left(\text{4}i-\text{1}\right)}-x_{\text{4}i}{)}^{\text{2}}+(x_{\left(\text{4}i-\text{2}\right)}-x_{\left(\text{4}i-\text{1}\right)}{)}^{\text{4}}+\text{1}\text{0}(x_{\left(\text{4}i-\text{3}\right)}-x_{\text{4}i}{)}^{\text{4}}] \end{array}$	$[-\text{4},\text{5}{]}^{\text{52}}$	0	UN
$\begin{array}{c} F_{\text{2}\text{0}}(X)=\sum_{i=\text{1}}^{n}\left\{\sum_{j=\text{0}}^{k}\left[a^{j}cos\left(\text{2}\pi b^{j}\left(x_{i}+\text{0}.\text{5}\right)\right)\right]\right\}-n\sum_{j=\text{0}}^{k}\left[a^{j}cos\left(\pi b^{j}\right)\right] \end{array}$ wherea=0.5,b=3,k=20	$[-\text{0}.\text{5},\text{0}.\text{5}{]}^{\text{n}}$	0	MS

#### 4.2.1. Test function result analysis.

LLSKSO incorporates multiple control strategies to strike a balance between exploring new solutions and leveraging current ones. Therefore, to verify the improvements made by LLSKSO, the comparison experiments of 20 benchmark test functions are given in this section. Table 3 gives the statistical results of LLSKSO and other comparison algorithms (RUN, STOA, POA, COA, SCSO, AOA, DBO, COA, CBOA, KSO and LLSKSO) for the benchmark functions with high dimensional benchmark functions (*pop* = 50, where, F10 is 52). The precision is 10^−10^, where mean, best, and variance are calculated after 30 runs of each algorithm, respectively. The end of the table shows the statistics of the mean, best, and variance results for each function.

**Table 3:** Test function results.

Function		RUN	STOA	CBOA	DBO	POA	AOA	NOA	SCSO	COA	KSO	LLSKSO
F1	M	1378.47	6.36E-11	0	0	1381.91	2.18E-01	0	0	0	0	0
	B	1076.21	1.23E-13	0	0	1201.79	7.39E-01	0	0	0	0	0
	V	16928.46	5.50E-20	0	0	8144.02	1.42E-02	0	0	0	0	0
F2	M	6.75E + 24	5.37E-09	0	0	1.25E + 26	0	0	0	0	0	0
	B	3.29E + 18	7.23E-11	0	0	2.74E + 18	0	0	0	0	0	0
	V	3.27E + 50	8.06E-17	0	0	1.81E + 53	0	0	0	0	0	0
F3	M	543158.17	0.73	0	1.41E-15	575020.36	0.01	0	0	0	672.9783	9.06E-02
	B	211108.01	0.01	0	0	183412.82	5.89E-07	0	0	0	3.45E + 04	9.06E-02
	V	30445	1.48	0	5.90E-29	49927488484	2.43E-08	0	0	0	582.3640	0
F4	M	94.544	2.79	0	0	94.39	218.66	0	0	0	0	0
	B	89.30	0.09	0	0	91.13	196	0	0	0	0	0
	V	4.76	68.07	0	0	2.74	60.71	0	0	0	0	0
F5	M	5566.90	48.60	2.99E-05	48.58	566526672	0	47.96	48.35	5.71E-07	41.2448	0
	B	378387603.7	47.15	2.13E-06	48.54	346631225.6	0	47.07	46.16	0	0.9366	0
	V	7.16E + 15	0.14	8.39E-09	0.08	1.02E + 16	0	0.16	0.67	9.78E-12	40.0085	0
F6	M	136684.46	0	0	0	135732.9	1.90E-04	0	0	0	0	0
	B	114470	0	0	0	111554	0	0	0	0	0	0
	V	74024732.67	0	0	0	100940216.9	0	0	0	0	0	0
F7	M	493.61	0.09	7.03E-06	6.99E-05	474.05	0.11	0.09	2.23E-05	6.90E-06	1.70E-02	8.76
	B	312.91	0.01	7.63E-07	1.33E-06	327.84	0.03	8.99E-05	8.36E-07	3.39E-07	1.41E-05	5.20
	V	3983.65	5.67E-05	2.71E-11	2.95E-09	4490.89	0.01	3.95E-07	6.63E-10	6.82E-11	1.04E-02	2.85
F8	M	−2359.61	−6537.00	−15050.04	−20924.68	−1726.65	0.06	−15133.36	−10250.09	−20949.14	−2.09E + 04	−2.09E + 04
	B	−4493.77	−8184.67	−17302.81	−20949.14	−3055.36	0.04	−17125.37	−11834.11	−20949.14	−2.09E + 04	−2.09E-14
	V	782198.92	389118.01	2452685.15	17862.71	454263.91	0.01	901818.49	668358.78	2.40E-05	1.39E-04	3.19E-24
F9	M	806.76	6.31	0	58.63	836.13	48.79	0	0	0	0	99.64
	B	682.04	1.19E-12	0	0	728.22	48.36	0	0	0	0	1.19E-25
	V	2837.99	120.48	0	4183.57	1600.24	0.04	0	0	0	0	2.17
F10	M	19.96	19.96	2.07E-15	8.88E-16	20.92	0.24	8.88E-16	8.88E-16	8.88E-16	0	8.88E-16
	B	19.96	19.96	8.88E-16	8.88E-16	20.69	9.19E-19	8.88E-16	8.88E-16	8.88E-16	0	8.88E-16
	V	5.22E-29	8.44E-07	2.90E-30	0	0.09	0.43	0	0	0	0	0
F11	M	1211.25	0.01	0	0.01	1194.25	−6137.14	0	0	0	5.26E-03	0
	B	984.80	5.44E-15	0	0	942.38	−6814.90	0	0	0	2.51E-05	0
	V	10560.98	0.06	0	6.02E-05	7172.68	228349.60	0	0	0	0	0
F12	M	1467609055	0.57	8.00E-12	0.20	1370038847	0	0.03	0.30	9.42E-33	0	0
	B	1087427741	0.37	2.43E-12	0.05	833086403.8	0	0.06	0.15	9.42E-33	0	0
	V	4.29E + 16	0.02	1.33E-23	0.01	5.89E + 16	0	1.59E-06	0.01	7.75E-96	0	0
F13	M	2754361926	4.18	1.81E-10	1.20	2632658907	0	0.16	4.64	1.35E-32	0	0
	B	1767785069	3.84	9.24E-11	0.61	2143813203	0	0.03	4.14	1.35E-32	0	0
	V	1.75E + 17	0.04	3.34E-21	0.14	8.03E + 16	0	0.03	0.02	3.10E-95	0	0
F14	M	245.20	56.03	18.233	13.56	244.9	920.76	0	97.4	0	0	−1.96E + 03
	B	219.00	11	1	0	209	723.94	0	78	0	0	−1.96E-13
	V	150.02	432.17	360.18	741.08	143.54	14801.59	0	110.11	0	0	1.18E-24
F15	M	31779.31	5.09E-12	0	0	32531.00	0.67	0	0	0	0	0
	B	22956.93	1.10E-16	0	0	25313.83	0.67	0	0	0	0	0
	V	15279021.55	2.40E-22	0	0	11966306.8	3.01E-11	0	0	0	0	0
F16	M	8.57118E + 11	3.81	0	17.04	9.88E + 11	2.07E-15	0	0	0	−1958.31	0
	B	5108162544	0.02	0	0	80099978893	8.88E-16	0	0	0	0	0
	V	7.29E + 23	50.28	0	4438.97	7.96E + 23	1.07E-29	0	0	0	−1958.31	0
F17	M	7226403.08	0.67	0.355	0.99	6976288.575	89.01	0.67	0.67	0.24	0	1.47E-12
	B	5159400.29	0.666	2.87E-08	0.943249365	4651051.2	15.58	0.66	0.67	0.24	0	0
	V	1.22955E + 12	0.01	0.11	0.06	1.29E + 12	6886.96	7.27E-06	0.03	1.56E-07	0	0
F18	M	2.23477E + 12	11.75	1.89	27.63	1.86E + 12	0.86	0.07	4.47	15.79	2.17E-02	0
	B	9961671860	0.50	0.04	0.018	789598075.4	0.75	0.01	0.08	2.74	4.12E-04	0
	V	5.21E + 24	109.19	9.85	3003.29	5.90E + 24	0.01	0.01	50.37	403.90	1.06E-02	0
F19	M	8839.56	5.58E-07	0	0	10339.74	4.91	0	0	0	4.91E-03	0
	B	2686.35	3.71E-13	0	0	5600.69	4.80	0	0	0	7.56E-07	0
	V	9028462.87	5.36E-12	0	0	12478756.12	0.04	0	0	9.48E-06	4.71E-03	0
F20	M	0	0	0.06	0	90.92	363.38	0	0	5.28E-02	0	0
	B	0	0	0	0	83.92	59.03	0	0	3.51E-02	0	0
	V	0	0	0.08	0	7.62	23809.31	0	0	2.43E-04	0	0
Total	M	0	11.00	9.00	12.00	8.00	7.00	9.00	3.00	15.00	15.00	**16.00**
	B	0	14.00	12.00	12.00	8.00	8.00	12.00	6.00	16.00	15.00	**18.00**
	V	0	12.00	10.00	11.00	8.00	7.00	10.00	3.00	15.00	15.00	**18.00**

From the results of the average value, LLSKSO obtained the theoretical optimal value in 16 (except F3, F7, F9, F14) test functions. Next, STOA and COA obtained 11 and 15 theoretical optimal values respectively. From Table 3, LLSKSO obtains the highest theoretical optimal values and is much higher than the theoretical optimal values of the initial KSO (3), which proves that the improvement of KSO is effective. The LLSKSO is also infinitely close to the theoretical values of the benchmark functions for F3 and F14. Therefore, from the results of the average value of LLSKSO, the improvement of LLSKSO is effective and better than other algorithms.

LLSKSO achieves 18 (except F3 and F7) theoretical optima in terms of optima. COA, which is in the second place, achieves only 16 optimal values. The performance of other MAs (especially KSO and RUN) is much lower than that of LLSKSO, and it is better than POA and RUN on the F3 and F7 test functions. This proves that LLSKSO is also excellent in the aspect of mean value, the effect performance.

In the aspect of mean value, LLSKSO also achieved the theoretical optimum under 18 (except F7, F9) test functions. The optimization effect of LLSKSO is not significant on the two test functions F7 and F9, but through other comparison algorithms, the other algorithms are also not effective.

Therefore, when LLSKSO is analyzed in terms of mean, best, and variance, the theoretical optimal values of the other comparison algorithms are all inferior to LLSKSO (especially KSO). This also proves that the improvement of LLSKSO is effective.

#### 4.2.2. Iteration curves for benchmark test functions.

**Fig 2:**
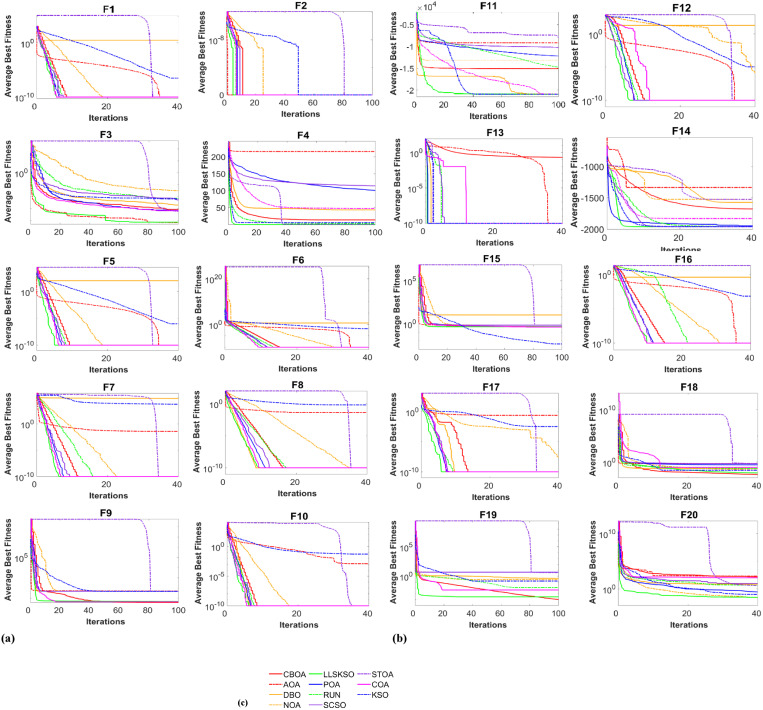
Iteration curve of each algorithm: (a) Convergence curves of the average best fitness for functions F1-F10, (b) Convergence curves of the average best fitness for functions F11-F20 and (c) Correspondence between curve colors and algorithms.

Whether fast convergence is a key step in proving the effectiveness of LLSKSO. Therefore, the convergence curves of LLSKSO algorithm on 20 high-dimensional benchmark test functions are compared with other MAs. Fig 2 can visually show the specific iteration curve.

As observed in Fig 2, the LLSKSO algorithm exhibits a quicker convergence rate compared to other algorithms across the majority of benchmark test functions. (F1-F2, F4-F13, F17, F19-F20, a total of 15) is superior, and the convergence speed is stronger than that of other MAs. In the convergence curve graph, LLSKSO mostly converges quickly (within the first 20%). The reason is the addition of the improvement mechanism enhances the convergence speed of LLSKSO. And LLSKSO has inflection points, which indicates that LLSKSO is constantly exploring and exploiting, preventing LLSKSO from falling into a local optimum. LLSKSO significantly enhances the accuracy of the algorithm and achieves a balance between exploration and exploitation for most benchmark functions. Therefore, the incorporation of the improvement mechanism effectively aids the algorithm’s exploration in the solution space, laying a solid foundation for subsequent exploitation.

For the remaining functions (F3, F14-F16, F18, 5 in total), LLSKSO searched the theoretical optimum. In F3, LLSKSO searched the theoretical optimum at about 50% of the iterations, and the AOA algorithm all reached the theoretical optimum at about 80% of the iterations. This shows that LLSKSO converges faster, but AOA converges with stronger accuracy. Therefore, the performance of LLSKSO and AOA is almost equal, but the rest of the compared algorithms are searching to the optimum, which shows the strong competitiveness of LLSKSO. In F14-F16 test functions, DBO, COA, and STOA converge faster. In F16, COA is almost equal to LLSKSO, and LLSKSO converges in the first 20% of the iterations, which proves the strong local search ability of LLSKSO. In F18, COA reaches the theoretical optimal value and LLSKSO does not reach the theoretical optimal value, but the convergence speed is faster, especially better than STOA.

Overall, LLSKSO is good and converges faster for most of the 20 functions tested. However, the convergence curves of STOA and AOA are irregular, which indicates that the algorithms are not as robust as LLSKSO and the other algorithms.

#### 4.2.3. CPU runtime.

**Fig 3:**
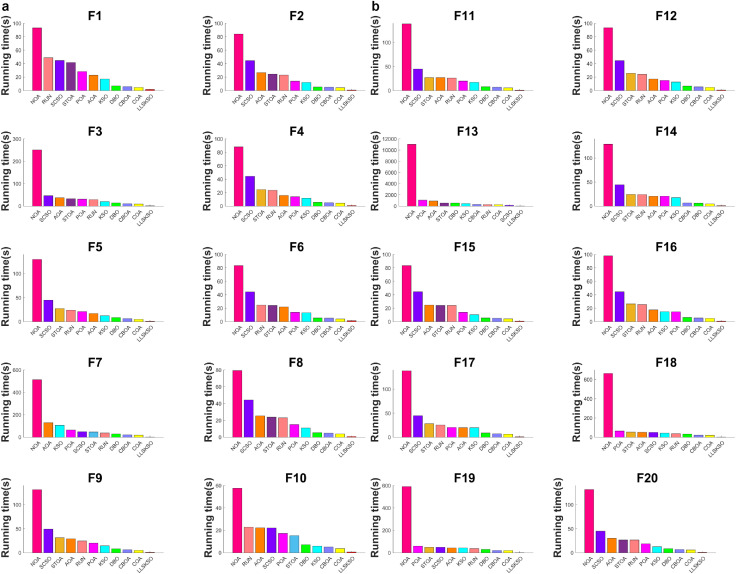
CPU runtime of each algorithm: (a) Running Time Comparison of Different Algorithms on F1-F10 and (b) Running Time Comparison of Different Algorithms on F11-F20.

CPU usage is an important metric for judging the performance of an algorithm. Fig 3 shows a histogram of the CPU usage of other algorithms in descending order. From the figure, it is evident that LLSKSO has the shortest time in all the 20 sets of test functions. Thus, under the same conditions, the CPU usage higher than LLSKSO. In practical applications, especially in complex systems, CPU usage is often a challenging issue to overcome. Therefore, the low CPU usage of LLSKSO presents a significant advantage in real-world applications, which clearly demonstrates that LLSKSO is an algorithm with markedly improved effectiveness.

#### 4.2.4. Paired statistical tests for benchmark test functions.

To evaluate the statistical significance of performance disparities between LLSKSO and other algorithms, therefore, Wilcoxon Signed Test (WST) was performed on LLSKSO using AOA, STOA, CBOA, SCSO, RUN, POA, NOA, DBO, COA and KSO, respectively. For this test, the null hypothesis posits that the performance of LLSKSO is not statistically different from that of other algorithms. “There is no difference in the median number of optimal solutions obtained by the LLSKSO algorithm and the comparison algorithms for the same test function”.

Table 4 is WST results. Where “*p*” represents the probability of the medians being the same, “*+*” indicates superiority at a level higher than the 95% significance level. “*-*” indicates inferiority at a level higher than the 95% significance level. “*=*” indicates that there is no significant difference between the two algorithms.

**Table 4:** WST results for LLSKSO and other algorithms on benchmark test functions. (*α* = 0.05).

Testing Function	LLSKSO/AOA				LLSKSO/STOA				LLSKSO/CBOA				LLSKSO/SCSO				LLKSO/RUN			
	p	T+	T-	win	p	T+	T-	win	p	T+	T-	win	p	T+	T-	win	p	T+	T-	win
F1	1	0	0	=	1	0	0	=	1	0	0	=	1	0	0	=	1	0	0	=
F2	1	0	0	=	1	0	0	=	1	0	0	=	1	0	0	=	1	0	0	=
F3	8.22E-06	0	351	+	1	0	0	=	1	0	465	+	1	0	465	+	1	0	465	+
F4	2.54E-06	0	435	+	1	0	0	=	1	0	465	+	1	0	465	+	1	0	465	+
F5	1.73E-06	0	465	+	1.73E-06	0	465	+	2.35E-06	462	3	–	1.73E-06	0	465	+	1.73E-06	0	465	+
F6	1	0	0	=	1	0	0	=	1	0	0	=	1	0	465	+	1	0	465	+
F7	1.72E-06	465	0	–	1.73E-06	0	465	–	1.73E-06	465	0	–	1.73E-06	0	465	–	1.73E-06	465	0	–
F8	1.73E-06	0	465	+	1.73E-06	0	465	+	1.73E-06	0	465	+	1.73E-06	0	465	+	1.73E-06	0	465	+
F9	1	0	0	=	1	0	0	=	1	0	0	=	1	0	465	+	1	0	465	+
F10	1	0	0	=	1.73E-06	0	465	+	1	0	0	=	1	0	0	=	1	0	465	+
F11	1.73E-06	0	465	+	1	0	0	=	1	0	465	+	1	0	465	+	1	0	465	+
F12	1.72E-06	0	465	+	1.73E-06	0	465	+	1	0	0	=	1.73E-06	0	465	+	0.000976563	0	66	+
F13	1.72E-06	0	465	+	1.73E-06	0	465	+	2.56E-06	0	435	+	1.73E-06	0	465	+	1.73E-06	0	465	+
F14	1.71E-06	0	465	+	1	0	0	=	5.54E-06	0	378	+	1.71E-06	0	465	+	1	0	465	+
F15	1	0	0	=	1	0	0	=	1	0	465	+	1	0	0	=	1	0	465	+
F16	1.72E-06	0	465	+	1.73E-06	0	465	+	1	0	0	=	1	0	0	=	1	0	465	+
F17	1.73E-06	0	465	+	1.73E-06	0	465	+	0.02	345	120	–	1.73E-06	0	465	+	4.32E-08	465	0	–
F18	1.72E-06	0	465	+	0.01	73	392	+	0.07	69	396	+	2.37E-05	27	438	+	0.278	179	286	+
F19	0.25	0	6	+	1	0	1	+	1	0	465	+	1	0	0	=	1	0	465	+
F20	1	0	0	=	1	0	0	=	3.79E-06	0	406	+	1	0	0	=	1	0	465	+
Total	12/7/1				10/10/0				10/7/3				12/7/1				16/2/2			
Testing Function	LLSKSO/POA				LLSKSO/NOA				LLSKSO/DBO				LLSKSO/COA				LLKSO/KSO			
	p	T+	T-	win	p	T+	T-	win	p	T+	T-	win	p	T+	T-	win	p	T+	T-	win
F1	1	0	0	=	1	0	0	=	1	0	0	=	1	0	0	=	1.44E-06	0	465	+
F2	1	0	0	=	1	0	0	=	1	0	0	=	1	0	0	=	1.47E-06	0	465	+
F3	1	0	465	+	1	0	0	=	0.25	0	6	+	1	0	0	=	1.55E-06	0	465	+
F4	1	0	465	+	1	0	465	+	1	0	0	=	1	0	0	=	1.39E-06	0	465	+
F5	1.73E-06	0	465	+	1.73E-06	0	465	+	1.73E-06	0	465	+	1.60E-06	0	465	+	1.72E-06	0	465	+
F6	1	0	0	=	1	0	465	+	1	0	0	=	1	0	0	=	1	0	0	=
F7	1.73E-06	465	0	–	1.73E-06	465	0	–	1.73E-06	465	0	–	1.73E-06	465	0	–	4.42E-05	34	431	+
F8	1.73E-06	0	465	+	1.73E-06	0	465	+	1.73E-06	0	465	+	2.02E-06	1.5	463.5	+	1.41E-06	0	465	+
F9	1	0	465	+	1	0	0	+	0.000244141	0	91	+	1	0	0	=	1.51E-06	0	465	+
F10	1	0	465	+	1	0	0	=	1	0	0	=	1	0	0	=	1.29E-06	0	465	+
F11	1	0	0	=	1	0	465	+	1	0	0	=	1	0	0	=	1.44E-06	0	465	+
F12	1.73E-06	0	465	+	1.73E-06	0	465	+	1.73E-06	0	465	+	1	0	0	=	1.38E-06	0	465	+
F13	1.73E-06	0	465	+	1.73E-06	0	465	+	1.73E-06	0	465	+	1	0	0	=	1.08E-06	0	465	+
F14	2.50E-06	0	435	+	1	0	465	+	1	0	0	=	1	0	0	=	1	0	0	=
F15	1	0	465	+	1	0	465	+	1	0	0	=	1	0	0	=	1.48E-06	0	465	+
F16	1	0	0	=	1	0	465	+	8.30E-06	0	351	+	1	0	0	=	1.61E-06	465	0	–
F17	1.73E-06	0	465	+	1.73E-06	0	465	+	1.73E-06	0	465	+	1.73E-06	465	0	–	1.65E-06	465	0	–
F18	1.73E-06	465	0	–	0.038	333	132	–	8.92E-05	42	423	+	1.92E-06	1	464	+	0.38	275	190	–
F19	1	0	0	=	1	0	465	+	1	0	0	=	1	0	465	+	1.58E-06	0	465	+
F20	1	0	0	=	1	0	465	+	1	0	0	=	1	0	0	=	1	0	0	=
Total	11/7/2				14/4/2				9/10/1					3/15/2			14/3/3			

Table 4 gives the total number of functions tested for LLSKSO and the comparison algorithms for the “*+*”, “*=*” and “*-*” cases in last row. It can be seen that STOA and LLSKSO perform similarly in benchmark function. LLSKSO slightly underperforms DBO and COA, but the difference is hardly significant. It is worth noting that LLSKSO is only inferior to other algorithms on a small subset of functions. Therefore, LLSKSO is still very competitive with other algorithms in two-by-two statistical tests for benchmark functions.

From the results of benchmark test functions, convergence curves, CPU occupation time and signed rank test. The performance of LLSKSO outperforms its comparative algorithms in a large part. Therefore, it can be seen that LLSKSO is highly competitive with benchmark test functions.

#### 4.2.5. Paired statistical tests for benchmark test functions.

The time complexity of LLSKSO can be approximated as O(G × N × D), where G is the number of generations, N is the population size, and D is the problem dimension. Due to its simplified structure and small population size (N = 10), LLSKSO achieves efficient computation with minimal parameter tuning.

### 4.3. Analysis of results of low-dimensional experiments

In this section, 30 benchmark test functions are used to validate and analyze the optimal effect of LLSKSO, which are classified into three types of variables. The three types are UN, MS, and MN, respectively. There are 6 UN and MS types and 18 MN types, respectively. The US type was not set in this subsection. This is because the US type is a single-peak separable variable, which has a simple form with only one peak and is not able to test the performance of LLSKSO. The number of runs is 30, and the mean (M), Best (B) and variance (V) of each algorithm’s optimization search results are counted (Table 5) [44].

**Table 5:** Benchmark test functions.

Functions	Range	${\text{f}}_{\text{min}}$	Type
$\begin{array}{c} F_{\text{2}\text{1}}(X)=(\frac{\text{1}}{\text{5}\text{0}\text{0}}+\sum_{j=\text{1}}^{\text{2}\text{5}}\frac{\text{1}}{j+\sum_{i=\text{1}}^{\text{2}}(x_{i}-a_{ij}{)}^{\text{6}}}{)}^{-\text{1}} \end{array}$ $[-\text{65}.\text{5},\text{65}.\text{5}{]}^{\text{2}}$	$[-\text{65}.\text{5},\text{65}.\text{5}{]}^{\text{2}}$	0.998	MS
$\begin{array}{c} F_{\text{2}\text{2}}=\sum_{i=\text{1}}^{\text{1}\text{1}}[a_{i}-\frac{x_{\text{1}}\left(b_{i}^{\text{2}}+b_{i}x_{\text{2}}\right)}{b_{i}^{\text{2}}+b_{i}x_{\text{3}}+x_{\text{4}}}{]}^{\text{2}} \end{array}$ $a_{i}=\{\text{0}.\text{1}\text{9}\text{5}\text{7},\text{0}.\text{1}\text{9}\text{4}\text{7},\text{0}.\text{1}\text{7}\text{3}\text{5},\text{0}.\text{1}\text{6},\text{0}.\text{0}\text{8}\text{4}\text{4},\text{0}.\text{0}\text{6}\text{2}\text{7},\text{0}.\text{0}\text{4}\text{5}\text{6},\text{0}.\text{0}\text{3}\text{4}\text{2},\text{0}.\text{0}\text{3}\text{2}\text{3},\text{0}.\text{0}\text{2}\text{3}\text{5},\text{0}.\text{0}\text{2}\text{4}\text{6}\}$ $b_{i}^{-\text{1}}=\left\{\text{0}.\text{2}\text{5},\text{0}.\text{5},\text{1},\text{2},\text{4},\text{6},\text{8},\text{1}\text{0},\text{1}\text{2},\text{1}\text{4},\text{1}\text{6}\right\}$	$[-\text{5},\text{5}{]}^{\text{4}}$	3.07E-04	MN
$F_{\text{2}\text{3}}(X)=\text{4}x_{\text{1}}^{\text{2}}-\text{2}.\text{1}x_{\text{1}}^{\text{4}}+\frac{\text{1}}{\text{3}}x_{\text{1}}^{\text{6}}+x_{\text{1}}x_{\text{2}}-\text{4}x_{\text{2}}^{\text{2}}+\text{4}x_{\text{2}}^{\text{4}}$	$[-\text{5},\text{5}{]}^{\text{2}}$	-1.0316	MN
$F_{\text{2}\text{4}}(X)=(x_{\text{2}}-\frac{\text{5}.\text{1}}{\text{4}\pi^{\text{2}}}x_{\text{1}}^{\text{2}}+\frac{\text{5}}{\pi }x_{\text{1}}-\text{6}{)}^{\text{2}}+\text{1}\text{0}(\text{1}-\frac{\text{1}}{\text{8}\pi })cosx_{\text{1}}+\text{1}\text{0}$	$[-\text{5},\text{15}{]}^{\text{2}}$	0.3979	MN
$\begin{array}{cc} F_{\text{2}\text{5}}(X)= & [\text{1}+(x_{\text{1}}+x_{\text{2}}+\text{1}{)}^{\text{2}}(\text{1}\text{9}-\text{1}\text{4}x_{\text{1}}+\text{3}x_{\text{1}}^{\text{2}}-\text{1}\text{4}x_{\text{2}}+\text{6}x_{\text{1}}x_{\text{2}}+\text{3}x_{\text{2}}^{\text{2}})]\times \;[\text{3}\text{0}+(\text{2}x_{\text{1}}-\text{3}x_{\text{2}}{)}^{\text{2}}\\ & (\text{1}\text{8}-\text{3}\text{2}x_{\text{1}}+\text{1}\text{2}x_{\text{1}}^{\text{2}}+\text{4}\text{8}x_{\text{2}}-\text{3}\text{6}x_{\text{1}}x_{\text{2}}+\text{2}\text{7}x_{\text{2}}^{\text{2}})] \end{array}$	$[-\text{5},\text{5}{]}^{\text{2}}$	3.00	MN
$\begin{array}{c} F_{\text{2}\text{6}}(X)=-\sum_{i=\text{1}}^{\text{4}}c_{i}exp(-\sum_{j=\text{1}}^{\text{3}}a_{ij}(x_{j}-p_{ij}{)}^{\text{2}}) \end{array}$ $c_{i}=\left\{\text{1},\text{1}.\text{2},\text{3},\text{3}.\text{2}\right\}$ $[\text{0},\text{1}{]}^{\text{3}}$ $[\text{0},\text{1}{]}^{\text{3}}$	$[\text{0},\text{1}{]}^{\text{3}}$	-3.8628	MN
$\begin{array}{c} F_{\text{2}\text{7}}(X)=-\sum_{i=\text{1}}^{\text{4}}c_{i}exp(-\sum_{j=\text{1}}^{\text{6}}a_{ij}(x_{j}-p_{ij}{)}^{\text{2}}) \end{array}$ $[\text{0},\text{1}{]}^{\text{6}}$ $[\text{0},\text{1}{]}^{\text{6}}$	$[\text{0},\text{1}{]}^{\text{6}}$	-3.32	MN
$\begin{array}{c} F_{\text{2}\text{8}}(X)=-\sum_{i=\text{1}}^{\text{5}}{\left[(X-a_{i})(X-a_{i}{)}^{T}+c_{i}\right]}^{-\text{1}} \end{array}$ $c_{i}=\left\{\text{0}.\text{1},\text{0}.\text{2},\text{0}.\text{2},\text{0}.\text{4},\text{0}.\text{4},\text{0}.\text{6},\text{0}.\text{3},\text{0}.\text{7},\text{0}.\text{5},\text{0}.\text{5}\right\}$ $c_{i}=\left\{\text{0}.\text{1},\text{0}.\text{2},\text{0}.\text{2},\text{0}.\text{4},\text{0}.\text{4},\text{0}.\text{6},\text{0}.\text{3},\text{0}.\text{7},\text{0}.\text{5},\text{0}.\text{5}\right\}$	$[\text{0},\text{10}{]}^{\text{4}}$	-10.1532	MN
$\begin{array}{c} F_{\text{2}\text{9}}(X)=-\sum_{i=\text{1}}^{\text{7}}{\left[(X-a_{i})(X-a_{i}{)}^{T}+c_{i}\right]}^{-\text{1}} \end{array}$ $a_{i}andc_{i}arethesameasF\text{3}\text{7}$	$[\text{0},\text{10}{]}^{\text{4}}$	-10.4029	MN
$\begin{array}{c} F_{\text{3}\text{0}}(X)=-{\sum_{i=\text{1}}^{\text{1}\text{0}}\left[(X-a_{i})(X-a_{i}{)}^{T}+c_{i}\right]}^{-\text{1}} \end{array}$ $a_{i}andc_{i}arethesameasF\text{3}\text{7}$	$[\text{0},\text{10}{]}^{\text{4}}$	-10.5364	MN
$F_{\text{3}\text{1}}(X)=(\text{1}.\text{5}-x_{\text{1}}+x_{\text{1}}x_{\text{2}}{)}^{\text{2}}+(\text{2}.\text{2}\text{5}-x_{\text{1}}+x_{\text{1}}x_{\text{2}}^{\text{2}}{)}^{\text{2}}+(\text{2}.\text{6}\text{2}\text{5}-x_{\text{1}}+x_{\text{1}}x_{\text{2}}^{\text{3}}{)}^{\text{2}}$	$[-\text{4}.\text{5},\text{4}.\text{5}{]}^{\text{2}}$	0	UN
$F_{\text{3}\text{2}}(X)=-cos\left(x_{\text{1}}\right)cos\left(x_{\text{2}}\right)exp\left(-{\left(x_{\text{1}}-\pi \right)}^{\text{2}}-{\left(x_{\text{2}}-\pi \right)}^{\text{2}}\right)$	[−100,100]	-1	UN
$F_{\text{3}\text{3}}(X)=\text{0}.\text{2}\text{6}\left(x_{\text{1}}^{\text{2}}+x_{\text{2}}^{\text{2}}\right)-\text{0}.\text{4}\text{8}x_{\text{1}}x_{\text{2}}$	$[-\text{10},\text{10}{]}^{\text{2}}$	0	UN
$[-\text{10},\text{10}{]}^{\text{2}}$	$[-\text{10},\text{10}{]}^{\text{4}}$	0	UN
$\begin{array}{c} F_{\text{3}\text{5}}(X)=\sum_{i=\text{1}}^{n}(x_{i}-\text{1}{)}^{\text{2}}-\sum_{i=\text{2}}^{n}x_{i}x_{i-\text{1}} \end{array}$	$[-\text{D},\text{D}{]}^{\text{6}}$	-50	UN
$\begin{array}{c} F_{\text{3}\text{6}}(X)=\sum_{i=\text{1}}^{n}(x_{i}-\text{1}{)}^{\text{2}}-\sum_{i=\text{2}}^{n}x_{i}x_{i-\text{1}} \end{array}$	$[-\text{D},\text{D}{]}^{\text{10}}$	-210	UN
$F_{\text{3}\text{7}}(X)=\left(x_{\text{1}}^{\text{2}}+\text{2}x_{\text{2}}^{\text{2}}\right)-\text{0}.\text{3}cos\left(\text{3}\pi x_{\text{1}}\right)-\text{0}.\text{4}cos(\text{4}\pi x_{\text{2}})+\text{0}.\text{7}$	$[-\text{100},\text{100}{]}^{\text{2}}$	0	MS
$F_{\text{3}\text{8}}(X)={\left(x_{\text{1}}+\text{2}x_{\text{2}}-\text{7}\right)}^{\text{2}}+{\left(\text{2}x_{\text{1}}+x_{\text{2}}-\text{5}\right)}^{\text{2}}$	[−10,10]	0	MS
$\begin{array}{c} F_{\text{3}\text{9}}=-\sum_{i=\text{1}}^{n}sin\left(x_{i}\right){\left(sin\left(\frac{ix_{i}^{\text{2}}}{\pi }\right)\right)}^{\text{2}m},m=\text{1}\text{0} \end{array}$	$[\text{0},\pi {]}^{\text{2}}$	−1.8013	MS
$\begin{array}{c} F_{\text{4}\text{0}}=-\sum_{i=\text{1}}^{n}sin\left(x_{i}\right){\left(sin\left(\frac{ix_{i}^{\text{2}}}{\pi }\right)\right)}^{\text{2}m},m=\text{1}\text{0} \end{array}$	$[\text{0},\pi {]}^{\text{5}}$	−4.68766	MS
$\begin{array}{c} F_{\text{4}\text{1}}=-\sum_{i=\text{1}}^{n}sin\left(x_{i}\right){\left(sin\left(\frac{ix_{i}^{\text{2}}}{\pi }\right)\right)}^{\text{2}m},m=\text{1}\text{0} \end{array}$	$[\text{0},\pi {]}^{\text{10}}$	−9.6602	MS
$F_{\text{4}\text{2}}=\text{0}.\text{5}+\frac{sin^{\text{2}}\left(\sqrt{x_{\text{1}}^{\text{2}}+x_{\text{2}}^{\text{2}}}\right)-\text{0}.\text{5}}{{\left(\text{1}+\text{0}.\text{0}\text{0}\text{1}\left(x_{\text{1}}^{\text{2}}+x_{\text{2}}^{\text{2}}\right)\right)}^{\text{2}}}$	$[-\text{100},\text{100}{]}^{\text{2}}$	0	MN
$F_{\text{4}\text{3}}=x_{\text{1}}^{\text{2}}+\text{2}x_{\text{2}}^{\text{2}}-\text{0}.\text{3}cos\left(\text{3}\pi x_{\text{1}}\right)cos\left(\text{4}\pi x_{\text{2}}\right)+\text{0}.\text{3}$	$[-\text{100},\text{100}{]}^{\text{2}}$	0	MN
$F_{\text{4}\text{4}}=x_{\text{1}}^{\text{2}}+\text{2}x_{\text{2}}^{\text{2}}-\text{0}.\text{3}cos\left(\text{3}\pi x_{\text{1}}+\text{4}\pi x_{\text{2}}\right)+\text{0}.\text{3}$	$[-\text{100},\text{100}{]}^{\text{2}}$	0	MN
$\begin{array}{c} F_{\text{4}\text{5}}=\left(\sum_{i=\text{1}}^{\text{5}}icos((i+\text{1})x_{\text{1}}+i\right)\times \left(\sum_{i=\text{1}}^{\text{5}}icos((i+\text{1})x_{\text{2}}+i\right) \end{array}$	$[-\text{10},\text{10}{]}^{\text{2}}$	-186.73	MN
$\begin{array}{c} F_{\text{4}\text{6}}=\sum_{k=\text{1}}^{n}{\left[\left(\sum_{i=\text{1}}^{n}x_{i}^{k}\right)-b_{k}\right]}^{\text{2}} \end{array}$	$[\text{0},\text{D}{]}^{\text{4}}$	0	MN
$\begin{array}{c} F_{\text{4}\text{7}}=-\sum_{i=\text{1}}^{m}c_{i}\left(exp\left(-\frac{\text{1}}{\pi }\sum_{j=\text{1}}^{n}{\left(x_{j}-a_{ij}\right)}^{\text{2}}\right)\right)\times cos\left(\pi \sum_{j=\text{1}}^{n}{\left(x_{j}-a_{ij}\right)}^{\text{2}}\right) \end{array}$	$[\text{0},\text{10}{]}^{\text{2}}$	-1.08	MN
$\begin{array}{c} F_{\text{4}\text{8}}=-\sum_{i=\text{1}}^{m}c_{i}\left(exp\left(-\frac{\text{1}}{\pi }\sum_{j=\text{1}}^{n}{\left(x_{j}-a_{ij}\right)}^{\text{2}}\right)\right)\times cos\left(\pi \sum_{j=\text{1}}^{n}{\left(x_{j}-a_{ij}\right)}^{\text{2}}\right) \end{array}$	$[\text{0},\text{10}{]}^{\text{5}}$	-1.5	MN
$\begin{array}{c} F_{\text{4}\text{9}}=-\sum_{i=\text{1}}^{m}c_{i}\left(exp\left(-\frac{\text{1}}{\pi }\sum_{j=\text{1}}^{n}{\left(x_{j}-a_{ij}\right)}^{\text{2}}\right)\right)\times cos\left(\pi \sum_{j=\text{1}}^{n}{\left(x_{j}-a_{ij}\right)}^{\text{2}}\right) \end{array}$	$[\text{0},\text{10}{]}^{\text{10}}$	-1.5	MN
$\begin{array}{c} F_{\text{5}\text{0}}=\sum_{i=\text{1}}^{n}{\left(A_{i}-B_{i}\right)}^{\text{2}}, \end{array}$ $\begin{array}{c} A_{i}=\sum_{j=\text{1}}^{n}a_{ij}sin\alpha_{j}+b_{ij}cos\alpha_{j}, \end{array}$ $\begin{array}{c} B_{i}=\sum_{j=\text{1}}^{n}a_{ij}sinx_{j}+b_{ij}cosx_{j} \end{array}$	$[-\pi ,\pi {]}^{\text{2}}$	0	MN

#### 4.3.1. Test function result analysis.

Table 6 indicates the results of LLSKSO and other algorithms on benchmark test functions (pop≤10). The accuracy is 10^-15^. Mean, Best and Var denote mean, optimum and variance respectively. The table shows the result statistics for different benchmark functions in last row. Overall, LLSKSO outperforms the other algorithms in all three aspects and obtains the best result in almost every benchmark function. This shows that LLSKSO has better performance in low dimensional benchmark test functions.

**Table 6:** Benchmark test function results.

Function		RUN	STOA	CBOA	DBO	POA	AOA	NOA	SCSO	COA	KSO	LLSKSO
F21	M	340.32	3.130	5.96	5.69	333.45	11.286	0.99	7.75	0.998	0.998	0.998
	B	18.52	0.998	0.99	0.99	11.14	2.982	0.99	0.99	0.998	0.998	0.998
	V	37207.57	7.964	16.00	20.32	29426.45	9.472	0	26.44	1.14E-22	0	0.998
F22	M	1.65	0.0035	0.02	0.04	1.09	0.0158	0.08	0.03	0.0004	0	0.000307
	B	0.01	0.0005	0.03	0.03	0.06	0.0003	0.03	0.03	0.0003	0	0.000307
	V	4.16	4.50E-05	0.01	0	4.51	0.0005	0	0	9.73E-08	0	8.76E-20
F23	M	−14.69	−1.0316	−1.03	−1.03	−13.58	−1.0316	−1.03	−1.03	−1.03	−1.03	−1.03
	B	−0.19	−1.0316	−1.03	−1.03	−0.25	−1.0316	−1.03	−1.03	−1.03	−1.03	−1.03
	V	−1064.34	5.33E-12	0	0	−501.21	7.18E-14	0	0	2.79E-07	−1.03	1.65E-31
F24	M	7.22	0.3986	0.40	0.72	7.58	0.43	0.40	0.40	0.41	0.39	0.39
	B	0.91	0.3978	0.40	0.40	0.90	0.40	0.40	0.40	0.39	0.39	0.39
	V	23.45	3.97E-08	0	0.63	22.97	0.002	0	0	0.0006	0	0.39
F25	M	14589.84	3.00	16.50	9.34	14677.23	24.01	3.00	3.00	4.51	3.00	3.00
	B	11.96	3.00	3.00	3.00	33.81	3	3.00	3.00	3.00	3.00	3.00
	V	3.00	6.64E-08	641.01	436.08	3.00	308.03	0	0	26.48	0	2.13E-28
F26	M	−2.89	−3.8550	−3.86	−3.77	−2.69	−3.84	−3.86	−3.86	−3.70	−3.86	−3.86
	B	−3.72	−3.8623	−3.86	−3.86	−3.84	−3.85	−3.86	−3.86	−3.86	−3.86	−3.86
	V	−0.33	1.94E-06	0	0.04	0.93	7.95E-05	0	0	0.01	1.09E-15	3.88E-30
F27	M	−1.01	−2.50	−3.38	−2.53	−0.92	−2.94	−3.30	−3.07	−1.97	−3.27	−3.25
	B	−2.67	−3.28	−3.32	−3.18	−2.75	−3.13	−3.32	−3.32	−3.11	−3.32	−3.32
	V	0.35	0.73	0	0.26	0.34	0.02	0	−0.12	0.16	0	−0.003
F28	M	−0.37	−1.36	−9.64	−7.04	−0.38	−3.28	−10.15	−4.36	−10.15	−10.54	−10.15
	B	−0.66	−10.1330	−10.15	−10.15	−1.42	−5.33	−10.15	−5.06	−10.15	−10.54	−10.15
	V	0.02	7.0422	2.42	5.27	0.06	1.65	0	−2.50	2.29E-13	0	1.87E-29
F29	M	0.39	−1.4624	−9.87	−7.37	−0.51	−3.32	−10.40	−4.44	−10.40	−10.38	−10.402
	B	0.60	−10.3739	−10.40	−10.40	−1.40	−8.10	−10.40	−10.40	−10.40	−10.38	−10.402
	V	0.01	6.8434	−2.63	−6.16	−0.06	4.11	0	−11.06	2.34E-10	0	1.41E-30
F30	M	0.53	−5.7481	−9.64	−6.79	−0.65	−4.15	−9.73	−6.33	−10.53	−8.32	−10.53
	B	0.96	−10.5183	−10.54	−10.53	−2.06	−7.41	−10.54	−10.54	−10.53	−8.98	−10.53
	V	0.03	17.2437	−4.20	−6.62	−0.12	2.81	−4.61	−9.39	1.89E-09	−9.25	4.68E-30
F31	M	8.18	1.05E-05	0.13	0	10.10	0.64	0	0	0.004	0	5.82E-30
	B	0.07	7.14E-07	0	0	0.04	0.0002	0	0	2.35E-09	0	3.08E-30
	V	103.73	8.70E-11	0.08	0	217.95	1.01	0	0	0.0001	0	1.65E-60
F32	M	0.05	−0.99	−1.00	−0.85	0	−0.13	−1.00	−1.00	−0.99	−1.00	−1.00
	B	0.71	−0.99	−1.00	−1.00	0	−0.99	−1.00	−1.00	−1	−1.00	−1.00
	V	0.03	2.24E-09	0	−0.10	0	0.11	0	0	2.76E-14	0	−1
F33	M	1.16	1.14E-66	0	0	1.25	0	0	0	0	0	0
	B	0.13	3.08E-92	0	0	0.14	0	0	0	0	0	0
	V	1.33	3.51E-131	0	0	1.47	0	0	0	0	0	0
F34	M	29483.21	2.24	0	10.09	20835.50	12.28	0	0.84	2.01E-05	0	0.04
	B	903.31	0.002	0	0.33	473.45	6.80	0	0	0	0	4.78E-11
	V	3393931412.00	5.27	0	89.81	851181483.30	7.90	0	0.49	1.21E-08	0	0.01
F35	M	739.51	−45.75	−50	−36.09	−828.48	−5.15	−50	−38.69	−36.99	−50.00	−50.00
	B	265.53	−49.99	−50	−46.84	−238.84	−9.07	−50	−50	−48.21	−50.00	−50.00
	V	34753.63	73.21	0	−43.93	−123170.86	3.09	0	−272.52	16.29	0	8.28E-26
F36	M	7777.13	−108.98	−210	−96.19	−14103.01	−7.20	−209.93	−66.02	−108.20	−210.00	−210.00
	B	3435.41	−197.11	−210	−160.32	−6827.33	−16.02	−210	−210	−167.35	−210.00	−210.00
	V	866063.69	2628.29	0	−363.57	−13310722.84	11.43	0.01	−2182.71	390.85	0	5.14E-22
F37	M	1718.89	0	0	0	2018.60	0	0	0	0	0.11	0
	B	16.01	0	0	0	123.85	0	0	0	0	0.12	0
	V	1707721.12	0	0	0	2495031.57	0	0	0	0	0.58	0
F38	M	44.10	4.16E-05	0	0.27	32.79	2.57	0	0	0.07	1.52E-11	4.21E-31
	B	2.79	2.54E-06	0	0	3.34	2.45E-07	0	0	9.57E-06	1.98E-02	0
	V	1088.32	1.32E-09	0	0.55	549.05	16.80	0	0	0.13	0	1.60E-61
F39	M	0.85	−1.34	−1.80	−1.80	−0.90	−1.65	−1.80	−1.63	−1.65	−1.803	−1.803
	B	1.71	−1.80	−1.80	−1.80	−1.68	−1.79	−1.80	−1.80	−1.80	−1.803	−1.803
	V	0.11	0.19	0	0	0.09	0.06	0	0.13	0.08	−1.803	−1.803
F40	M	1.35	−2.91	−4.63	−3.62	−1.45	−2.14	−4.68	−3.90	−3.08	−4.69	−4.59
	B	2.20	−4.62	−4.69	−4.48	−2.49	−2.74	−4.69	−4.69	−4.08	−4.69	−4.68
	V	0.22	0.61	0	−0.22	−0.23	0.09	0	−0.31	0.14	0	−4.68
F41	M	2.39	−4.28	−9.32	−5.30	−2.36	−3.47	−9.56	−6.19	−4.59	−9.66	−9.14
	B	4.71	−6.00	−9.61	−6.93	−3.06	−4.17	−9.66	−8.25	−5.16	−9.35	−9.57
	V	0.50	0.82	−0.03	−0.57	−0.19	0.14	0.01	−1.56	0.11	0	0.05
F42	M	0.42	0.002	0	0.01	0.38	0	0	0	0	0.01	0
	B	0.07	0	0	0	0.13	0	0	0	0	0.01	0
	V	0.01	1.75E-05	0	0	0.01	0	0	0	0	0	0
F43	M	1800.47	0	0	0	2027.98	0	0	0	0	1.24	0
	B	35.77	0	0	0	41.68	0	0	0	0	1.5	0
	V	2604220.09	0	0	0	2786456.01	0	0	0	0	0.12	0
F44	M	1444.56	0	0	0	1559.25	0	0	0	0	0	0
	B	6.08	0	0	0	75.36	0	0	0	0	0	0
	V	1070781.45	0	0	0	2415920.57	0	0	0	0	0	0
F45	M	36.65	−186.61	−186.73	−186.51	−33.33	−124.99	−186.73	−186.73	−184.98	−186.73	−186.73
	B	162.43	−186.73	−186.73	−186.73	−76.02	−186.73	−186.73	−186.73	−186.73	−186.73	−186.73
	V	1091.35	0.05	0	0.77	−334.71	2063.71	0	0	10.42	−186.73	−183.73
F46	M	517.19	45.40	0.07	1.25	381.19	9.58	0	2.29	0.26	0	0.01
	B	1.58	0.02	0	0	0.46	0.58	0	0	0.0008	0	0
	V	638442.58	3738.59	0.02	14.36	211483.37	378.53	0	37.15	0.20	0	0
F47	M	0.21	−0.90	−1.06	−1.05	−0.17	−1.04	−1.08	−0.98	−0.86	−1.08	−1.08
	B	0.96	−1.08	−1.08	−1.08	−0.91	−1.07	−1.08	−1.08	−1.07	−1.08	−1.08
	V	0.07	0.10	−0.01	0	0.06	0.0005	0	−0.07	0.12	0	9.01E-32
F48	M	0.02	−0.27	−0.72	−0.67	−0.04	−0.64	−1.35	−0.73	−0.35	−1.25	−1.5
	B	0.20	−1.49	−1.50	−1.40	−0.80	−0.79	−1.50	−1.50	−0.79	−1.03	−1.5
	V	0	0.11	−0.10	−0.10	−0.02	0.02	−0.10	−0.18	0.07	−0.21	−1.5
F49	M	0	−0.02	0.06	0	0	−0.15	0.61	0.32	−0.0007	0	0
	B	0	−0.18	0.22	0.05	0	−0.44	1.50	1.50	−0.004	0	0
	V	0	0.002	0	0	0	0.013	0.10	0.11	2.74E-06	0	0
F50	M	2266.89	47.10	23.51	358.12	5453.66	521.01	0	0	297.29	0	1.78E-27
	B	255.02	0.0002	0	0	130.48	1.69E-05	0	0	2.96	0	0
	V	1385288.58	32010.88	16581.37	261966.11	105376760.70	803478	0	0	85507.10	0	2.27E-54
Total	M	0	6	14	4	0	5	20	10	10	14	**25**
	B	0	11	25	14	0	5	26	20	16	18	**29**
	V	1	6	7	4	0	5	10	5	5	17	**18**

In the aspect of Mean, LLSKSO achieves the theoretical optimum on 25 benchmark functions. NOA follows closely with the optimum on 20 benchmark functions. For other benchmark functions, LLSKSO did not achieve the optimal results, but by comparing and analyzing the results with the other algorithms, LLSKSO is closer to the theoretical values, especially than RUN.

The variance represents the robustness of the algorithm. LLSKSO achieved best result on 18 benchmark functions and ranked first. It is followed by KSO. While all other compared algorithms are below 10. This shows that LLSKSO consistently outperforms the comparison algorithm in robustness and stability.

As far as the best results are concerned, LLSKSO achieves the theoretical optimum on 29 benchmark functions, while KSO only 18. LLSKSO only obtained inferior results on a very small number of benchmark functions, but it is also close to the theoretical optimum. This proves that the improvement of LLSKSO is very obvious on low-dimensional test functions.

In conclusion, LLSKSO performs well in function testing in benchmark functions. It proves that LLSKSO can be used as an effective optimization algorithm.

#### 4.3.2 Iteration curves of benchmark functions.

**Fig 4:**
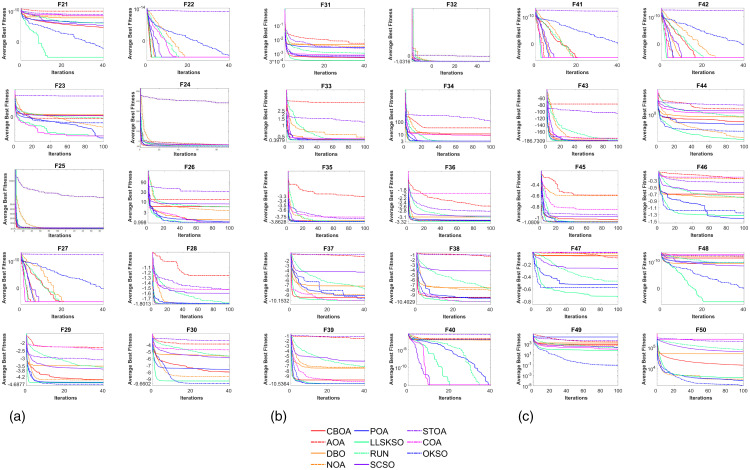
Iteration curve of benchmark function: (a) Convergence curves of the average best fitness for functions F21-F30, (b) Convergence curves of the average best fitness for functions F31-F40 and (c) Convergence curves of the average best fitness for functions F41-F50.

Fig 4 indicates the iteration curves for the benchmark functions. The line color is the same as in Fig 2. Similar to the iterative curves of the high-dimensional benchmark test functions, F21-F22, F24, F26-F31, F33-F39 and F41-F49 find the optimal value in the exploration phase. F23, F25, F32, F42 and F40 also around the optimal value. It is worth noting that STOA performs poorly in most of the iterative curves.

The convergence curves in Fig 4 demonstrate LLSKSO’s accelerated optimization process. Compared with state-of-the-art methods [48–52], our algorithm achieves superior convergence in 80% of test functions (especially for dim > 50), with the notable advantage of eliminating manual parameter tuning – a critical limitation in [49,51]. This enhances generalizability for industrial applications as shown in Section 5.

In summary, LLSKSO converges very rapidly, which shows that LLSKSO also converges very quickly.

#### 4.3.3. CPU runtime of benchmark test functions.

**Fig 5:**
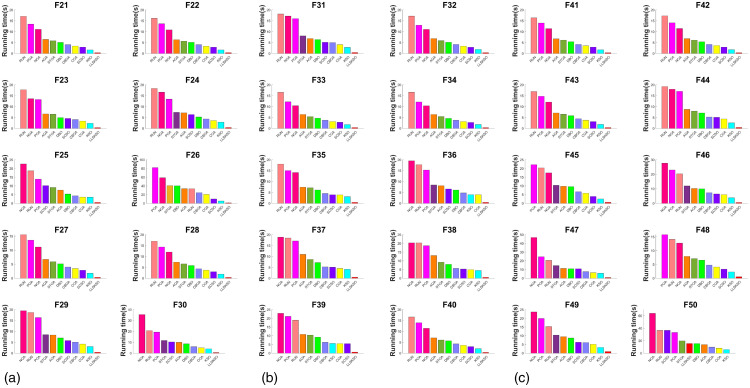
CPU usage of benchmark function: (a) CPU Usage during the Execution of Benchmark Functions F21-F30, (b) CPU Usage during the Execution of Benchmark Functions F31-F40 and (c) CPU Usage during the Execution of Benchmark Functions F41-F50.

Fig 5 indicates the histograms of CPU usage of other algorithms. It can be seen from that RUN, POA, and NOA occupy more CPU usage. AOA and STOA is next best performer. DBO, CBOA, COA, and SCSO have similar time shares and decrease in this way. KSO has an even smaller proportion. LLSKSO (with a slightly higher F50) occupy less CPU usage. This is due to the fact that LLSKSO requires more functions to be computed per iteration. With the same number of runs as the high-dimensional benchmark test functions, the CPU usage by LLSKSO in the low-dimensional benchmark test functions is almost insignificantly different from the high-dimensional ones, and the results obtained are relatively good. Therefore, from the above analysis and the results shown in the figures, LLSKSO still maintains the lowest CPU usage.

#### 4.3.4. Low-dimensional test function signed-rank test.

Table 7 shows the WST results of LLSKSO compared to the other algorithms under the benchmark functions. The total number of (+/ = /-) for LLSKSO versus the comparison algorithm in last row. The data shows that LLSKSO outperforms other algorithms.

**Table 7:** WST results for LLSKSO and other algorithms on benchmark test functions. (*α* = 0.05).

Testing Function	LLSKSO/STOA				LLSKSO/CBOA				LLSKSO/SCSO				LLSKSO/RUN				LLKSO/POA			
	p	T+	T-	win	p	T+	T-	win	p	T+	T-	win	p	T+	T-	win	p	T+	T-	win
F21	1.72E-06	0	465	+	1.73E-06	0	465	+	1	0	0	=	0.125	0	10	+	1	0	0	=
F22	1	0	0	=	1	0	0	=	1	0	0	=	1	0	0	=	1	0	0	=
F23	1.72E-06	0	465	+	7.69E-06	450	15	–	0.87	240	225	–	001	64	401	+	1.73E-06	465	0	–
F24	1.72E-06	0	465	+	1.73E-06	0	465	+	0.03	0	21	+	1.73E-06	0	465	+	0.0625	0	15	+
F25	1.72E-06	0	465	+	1.73E-06	0	465	+	1.73E-06	0	465	+	1.73E-06	0	465	+	1.73E-06	0	465	+
F26	1.72E-06	0	465	+	0.5	0	3	+	1.68E-05	0	300	+	7.88E-06	0	351	+	002	0	153	=
F27	1	0	0	=	1	0	0	=	1	0	0	=	1	0	0	=	1	0	0	=
F28	1.72E-06	0	465	+	1.73E-06	0	465	+	0.06	0	15	+	8.24E-06	0	351	+	1	0	0	=
F29	1.72E-06	0	465	+	1.73E-06	0	465	+	1.73E-06	0	465	+	1.73E-06	0	465	+	1.73E-06	0	465	+
F30	1.72E-06	0	465	+	1.73E-06	0	465	+	1.73E-06	0	465	+	1.73E-06	0	465	+	1.73E-06	0	465	+
F31	1.72E-06	0	465	+	1.73E-06	0	465	+	2.56E-06	0	435	+	0.855	195	211	–	0.37	210	141	–
F32	1.72E-06	0	465	+	1.73E-06	0	465	+	1	0	0	=	1	0	0	=	1	0	0	=
F33	1.72E-06	0	465	+	1.73E-06	0	465	+	1	0	0	=	004	0	78	+	1	0	0	=
F34	1.72E-06	0	465	+	1.73E-06	0	465	+	0.01	0	28	+	1.73E-06	0	465	+	1	0	0	=
F35	1.72E-06	0	465	+	1.73E-06	0	465	+	2.56E-06	0	435	+	1.73E-06	0	465	+	0.5	0	3	+
F36	1.72E-06	0	465	+	1.73E-06	0	465	+	1.24E-05	20	445	+	4.71E-06	10	455	+	6.10E-05	0	120	+
F37	1.73E-06	0	465	+	1.73E-06	0	465	+	1.73E-06	0	465	+	1.73E-06	0	465	+	0.0625	0	15	+
F38	1.73E-06	0	465	+	1.73E-06	0	465	+	1.73E-06	0	465	+	1.73E-06	0	465	+	0.5	0	3	+
F39	1.72E-06	0	465	+	1.73E-06	0	465	+	2.56E-06	0	435	+	1.73E-06	0	465	+	0.25	0	6	+
F40	1	0	0	=	1	0	0	=	1	0	0	=	1	0	0	=	1	0	0	=
F41	1	0	0	=	1	0	0	=	1	0	0	=	1	0	0	=	1	0	0	=
F42	1	0	0	=	1	0	0	=	1	0	0	=	1	0	0	=	1	0	0	=
F43	1.72E-06	0	465	+	1.73E-06	0	465	+	1	0	0	=	2.55E-06	0	435	+	1	0	1	+
F44	1.72E-06	0	465	+	1.92E-06	1	464	+	4.29E-06	9	456	+	0.09	152	313	+	01	77	388	+
F45	1.72E-06	0	465	+	1.73E-06	0	465	+	001	0	190	+	004	0	78	+	1	0	0	=
F46	1.72E-06	0	465	+	1.73E-06	0	465	+	3.90E-06	4	431	+	2.59E-06	4	461	+	6.28E-06	1	377	+
F47	01	75	390	+	1.73E-06	0	465	+	1.73E-06	0	465	+	1	74	391	+	0.974	219	216	–
F48	1.72E-06	0	465	+	1.73E-06	0	465	+	1	0	1	+	1.73E-06	0	465	+	0.25	0	6	+
F49	1.72E-06	0	465	+	1.73E-06	0	465	+	1.36E-05	21	444	+	1.73E-06	0	465	+	1.73E-06	0	465	+
F50	1.72E-06	0	465	+	1.73E-06	0	465	+	3.18E-06	6	459	+	1.73E-06	0	465	+	1.73E-06	0	465	+
Total	25/5/0				24/5/1				20/9/1				24/6/0				15/12/3			
Testing Function	LLSKSO/POA				LLSKSO/NOA				LLSKSO/DBO				LLSKSO/COA				LLKSO/KSO			
	p	T+	T-	win	p	T+	T-	win	p	T+	T-	win	p	T+	T-	win	p	T+	T-	win
F21	1	0	0	=	1	0	0	=	03	0	45	+	1.73E-06	0	465	+	1	0	0	=
F22	1	0	0	=	1	0	0	=	1	0	0	=	1	0	0	=	1	0	0	=
F23	1.44E-06	465	0	–	1.67E-06	465	0	–	1.92E-06	1	464	+	1.64E-06	465	0	–	6.28E-06	13	452	+
F24	1.73E-06	0	465	+	0.125	0	10	+	1.73E-06	0	465	+	1.73E-06	0	465	+	3.10E-06	0	406	+
F25	1.73E-06	0	465	+	1.73E-06	0	465	+	1.73E-06	0	465	+	1.73E-06	0	465	+	1.39E-06	0	465	+
F26	1	0	0	=	1	0	465	+	1	0	1	+	1	0	0	=	1	0	0	=
F27	1	0	0	=	1	0	465	+	1	0	0	=	1	0	0	=	009	0	66	+
F28	1	0	465	+	1	0	0	=	00196437	0	171	+	1.73E-06	0	465	+	1	0	0	=
F29	1.73E-06	0	465	+	0.5	0	3	+	1.73E-06	0	465	+	1.73E-06	0	465	+	1.98E-06	0	435	+
F30	1.73E-06	0	465	+	1.73E-06	0	465	+	1.73E-06	0	465	+	1.73E-06	0	465	+	02	87	378	+
F31	02	276	49	–	9.42E-06	325	0	–	1.73E-06	0	465	+	1.73E-06	0	465	+	1.70E-06	0	465	+
F32	1	0	0	=	1	0	465	+	1	0	0	=	1.73E-06	0	465	+	1	0	465	+
F33	1	0	465	+	1	0	465	+	004	0	78	+	1.73E-06	0	465	+	1	0	0	=
F34	1	0	465	+	1	0	465	+	0.25	0	6	+	1.73E-06	0	465	+	009	0	66	+
F35	1	0	465	+	1	0	465	+	1.73E-06	0	465	+	1.73E-06	0	465	+	001	0	171	+
F36	2.84E-05	29	436	+	0.125	0	10	+	1.73E-06	0	465	+	1.73E-06	0	465	+	002	1	104	+
F37	1	0	0	=	1	0	0	=	1.73E-06	0	465	+	1.73E-06	0	465	+	1.61E-06	0	465	+
F38	1	0	0	=	1	0	0	=	1.73E-06	0	465	+	1.70E-06	0	465	+	1.27E-06	0	465	+
F39	1	0	465	+	0.125	0	10	+	1.73E-06	0	465	+	1.73E-06	0	465	+	1.51E-06	0	465	+
F40	1	0	465	+	1	0	465	+	1.31E-05	0	190	+	1	0	0	=	1.48E-06	0	465	+
F41	1	0	465	+	1	0	65	+	1	0	0	=	1	0	0	=	004	0	78	+
F42	1	0	465	+	1	0	32	+	1	0	0	=	1	0	0	=	1	0	0	=
F43	1	0	0	=	1	0	0	=	8.86E-05	0	210	+	1.73E-06	0	465	+	1.64E-06	0	465	+
F44	1.73E-06	465	0	–	0.057070218	325	140	–	2.35E-06	3	462	+	1.73E-06	0	465	+	0.19	170	295	+
F45	1	0	0	=	1	0	0	=	03	0	45	+	1.73E-06	0	465	+	01	0	55	+
F46	2.37E-05	27	438	+	0.01953125	3	42	+	1.73E-06	0	465	+	1.73E-06	0	465	+	004	12	198	+
F47	04	403.5	61.5	–	0.688328063	202	213	+	1.73E-06	0	465	+	1.73E-06	0	465	+	0.5	260	205	+
F48	1	0	0	–	1	0	0	=	001	0	190	+	1.73E-06	0	465	+	009	0	66	+
F49	3.52E-06	7	458	+	0.032045566	25	75	+	1.73E-06	0	465	+	1.73E-06	0	465	+	1.66E-06	0	465	+
F50	8.47E-06	16	449	+	1.73E-06	0	465	+	1.73E-06	0	465	+	1.73E-06	0	465	+	2.82E-05	29	436	+
Total	17/9/4				19/8/3				25/5/0				24/6/0				24/6/0			

From the results of low-dimensional test functions, convergence curves, CPU occupation time and WRST, LLSKSO also outperforms its comparison algorithms (especially KSO). Therefore, it can be seen that LLSKSO is also very competitive under the benchmark functions.

In summary, LLSKSO performs very well on both high and benchmark functions. Therefore, it can be foreseen that the improvement of LLSKSO is effective and can be used as an optimizer on engineering problems.

## 5. Carbon fiber drafting ratio optimization problem

### 5.1. Carbon fiber background

**Fig 6:**
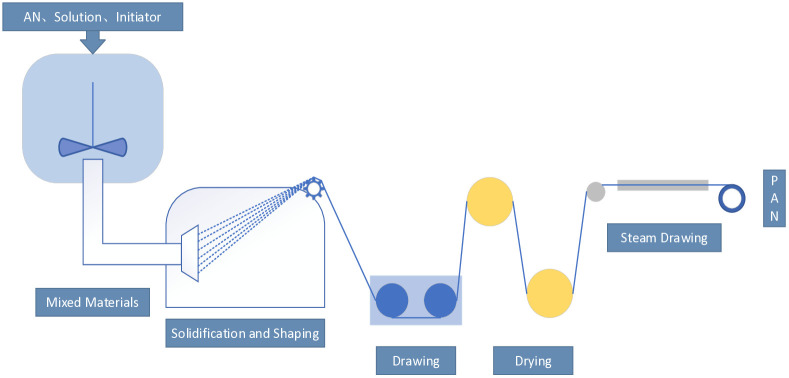
Flow chart of carbon fiber production.

Carbon fiber [57] is a high-strength, high-temperature resistant specialty fiber. Because of its excellent performance, it is called “special fiber” [58]. As shown in Fig 6, carbon fiber contains many complex production links, in which the raw silk is the preparation of high-performance carbon fiber prerequisite [59]. Drafting is an important step in the production process of filament, and the distribution of drafting ratio will directly affect the quality of carbon fiber filament. Therefore, the rational allocation of the drafting ratio will become an issue worth studying.

Draft process is an important step to reduce the density and improve the strength of carbon fiber filament. The drafting ratio, an important control parameter in the carbon fiber drafting process, is the most important factor affecting the mechanical properties of carbon fiber filament. Generally speaking, the drafting process of carbon fiber tow is divided into three levels, and each level of drafting includes a corresponding number of steps. Specifically, air drafting and solidification bath drafting are primary drafting processes. Hot water drafting and boiling water drafting are secondary drafting processes. Dry heat drafting and steam drafting are tertiary drafting processes.

The six drafting ratio parameters of carbon fiber are as follows: $r_{\text{1}}$ is the nozzle drafting ratio, $r_{\text{2}}$ is the air drafting ratio, $r_{\text{3}}$ is the solidification bath drafting ratio, $r_{\text{4}}$ is the hot water drafting ratio, $r_{\text{5}}$ is the boiling water drafting ratio, and $r_{\text{6}}$ is the tertiary drafting ratio [57]. Therefore, in this paper, the multi-step drafting of carbon fiber primary filaments is taken as the research objective, and the key parameters (line density, strength, elongation at break) are optimized by using LLSKSO, so as to further solve the optimal carbon fiber drafting ratio and drafting multiplier.

### 5.2. Mathematical modeling of carbon fibers

Unrestricted stretching will inevitably lead to breakage of the carbon fiber filaments. Balancing the distribution of each step is the only way to effectively prevent the breakage of the primary filament. The relationship between the performance parameters of carbon fiber filaments and the drafting ratio can be shown by Eq. (15).


$${(\rho }_{L}(i),T_{g}(i)),E_{L}(i)=f(r_{\text{1}i},\ldots r_{ki},\ldots r_{ni})$$ (15)

where f(*) is defined as a function. $r_{ki}$ denotes the *k*th step draft ratio of the carbon fiber filament. $\rho_{L}(i)$ denotes the linear density of the carbon fiber filament. $T_{g}(i)$ denotes the strength of the carbon fiber filament. $E_{L}(i)$ denotes the elongation at break of the carbon fiber.

Regarding the relationship between the three raw filament property indexes, namely, carbon fiber raw filament line density, strength, and elongation at break, and the drafting ratios of each step. We draw on the equations defined in the literature [60], and the mathematical model is shown in Eq. (16).


$$s_{i}=f\left(r_{i}\right)=a\times \left(r_{i}^{b}\right)\times \text{exp}\left(\frac{c}{r_{i}}\right),r_{i}\geq \text{1}.\text{0}$$ (16)

where $r_{i}$ is the drafting ratio. $s_{i}$ is the value of the performance parameter obtained in the *i*-th experiment. a,b,c are constants.

Based on the different roles of each drafting stage, the importance of each stretching stage was scored [57] and calculate its specific weight value. According to the derived weight values, the relationship between the draft ratio $r_{i}$ and the line density $S_{\text{1}i}$, strength $S_{\text{2}i}$ and Elongation at break $S_{\text{3}i}$ is fitted as shown in Eq. (17).


$$S_{\text{1}i}=f_{\text{1}}\left(r_{i}\right)=q_{i}*(\text{9}\text{4}.\text{7}\text{2}r_{i}^{-\text{1}.\text{3}\text{7}}exp\left(\frac{-\text{3}.\text{0}\text{5}}{r_{i}}\right)$$

$$S_{\text{2}i}=f_{\text{2}}\left(r_{i}\right)=q_{i}*(\text{3}\text{4}.\text{4}\text{8}r_{i}^{-\text{3}.\text{0}\text{8}}\text{exp}\left(\frac{-\text{8}.\text{5}\text{4}}{r_{i}}\right)$$ (17)

$$S_{\text{3}i}=f_{\text{3}}\left(r_{i}\right)=q_{i}*(\text{0}.\text{3}\text{7}r_{i}^{\text{0}.\text{9}\text{9}}\text{exp}\left(\frac{\text{1}\text{4}.\text{2}\text{8}}{r_{i}}\right)$$

$$\text{1}\leq r_{\text{1}}\leq \text{4}.\text{5};\text{1}\leq r_{\text{2}}\leq \text{2};\text{1}\leq r_{\text{3}}\leq \text{2};\text{2}\leq r_{\text{4}}\leq \text{6};\text{1}\leq r_{\text{5}}\leq \text{1}.\text{3};\text{1}\leq r_{\text{6}}\leq \text{2}.\text{5};$$

where $q_{i}$ is the weight value of the draft ratio.

In order to fit the relationship between the draft ratio $r_{i}$ and the line density $S_{\text{1}i}$ and intensity $S_{\text{2}i}$ and elongation at rupture $S_{\text{3}i}$, it is also necessary to solve for $q_{i}$. The value of $q_{i}$ is obtained by scoring the importance of the material experts for the different roles of each stretching stage as follows [61].


$$\left[\begin{array}{c} \begin{array}{c} q_{\text{1}}=\text{0}.\text{3}\text{1}\text{0}\text{2}\\ q_{\text{2}}=\text{0}.\text{1}\text{7}\text{0}\text{3}\\ q_{\text{3}}=\text{0}.\text{0}\text{8}\text{9}\text{5} \end{array}\\ q_{\text{4}}=\text{0}.\text{1}\text{7}\text{0}\text{3}\\ q_{\text{5}}=\text{0}.\text{1}\text{7}\text{0}\text{3}\\ q_{\text{6}}=\text{0}.\text{0}\text{8}\text{9}\text{5} \end{array}\right]$$

Therefore, the objective function is as follows.

Line Density:


$$\mathrm{\backslash footnotesize}\begin{array}{cc} f_{\text{1}}\left(r_{i}\right)= & \text{0}.\text{3}\text{1}\text{0}\text{2}*\text{9}\text{4}.\text{7}\text{2}r_{\text{1}}^{-\text{1}.\text{3}\text{7}}exp\left(\frac{-\text{3}.\text{0}\text{5}}{r_{\text{1}}}\right)+\text{0}.\text{1}\text{7}\text{0}\text{3}*\text{9}\text{4}.\text{7}\text{2}r_{\text{2}}^{-\text{1}.\text{3}\text{7}}exp\left(\frac{-\text{3}.\text{0}\text{5}}{r_{\text{2}}}\right)+\text{0}.\text{0}\text{8}\text{9}\text{5}*\text{9}\text{4}.\text{7}\text{2}r_{\text{3}}^{-\text{1}.\text{3}\text{7}}exp\left(\frac{-\text{3}.\text{0}\text{5}}{r_{\text{3}}}\right)\\ & +\text{0}.\text{1}\text{7}\text{0}\text{3}*\text{9}\text{4}.\text{7}\text{2}r_{\text{4}}^{-\text{1}.\text{3}\text{7}}exp\left(\frac{-\text{3}.\text{0}\text{5}}{r_{\text{4}}}\right)+\text{0}.\text{1}\text{7}\text{0}\text{3}*\text{9}\text{4}.\text{7}\text{2}r_{\text{5}}^{-\text{1}.\text{3}\text{7}}exp\left(\frac{-\text{3}.\text{0}\text{5}}{r_{\text{5}}}\right)+\text{0}.\text{0}\text{8}\text{9}\text{5}*\text{9}\text{4}.\text{7}\text{2}r_{\text{6}}^{-\text{1}.\text{3}\text{7}}exp\left(\frac{-\text{3}.\text{0}\text{5}}{r_{\text{6}}}\right) \end{array}$$

Intensity:


$$\begin{array}{cc} f_{\text{2}}\left(r_{i}\right)= & \text{0}.\text{3}\text{1}\text{0}\text{2}*\text{3}\text{4}.\text{4}\text{8}r_{\text{1}}^{-\text{3}.\text{0}\text{8}}\text{exp}\left(\frac{-\text{8}.\text{5}\text{4}}{r_{\text{1}}}\right)+\text{0}.\text{1}\text{7}\text{0}\text{3}*\text{3}\text{4}.\text{4}\text{8}r_{\text{2}}^{-\text{3}.\text{0}\text{8}}\text{exp}\left(\frac{-\text{8}.\text{5}\text{4}}{r_{\text{2}}}\right)+\text{0}.\text{0}\text{8}\text{9}\text{5}*\text{3}\text{4}.\text{4}\text{8}r_{\text{3}}^{-\text{3}.\text{0}\text{8}}\text{exp}\left(\frac{-\text{8}.\text{5}\text{4}}{r_{\text{3}}}\right)\\ & +\text{0}.\text{1}\text{7}\text{0}\text{3}*\text{3}\text{4}.\text{4}\text{8}r_{\text{4}}^{-\text{3}.\text{0}\text{8}}\text{exp}\left(\frac{-\text{8}.\text{5}\text{4}}{r_{\text{4}}}\right)+\text{0}.\text{1}\text{7}\text{0}\text{3}*\text{3}\text{4}.\text{4}\text{8}r_{\text{5}}^{-\text{3}.\text{0}\text{8}}\text{exp}\left(\frac{-\text{8}.\text{5}\text{4}}{r_{\text{5}}}\right)+\text{0}.\text{0}\text{8}\text{9}\text{5}*\text{3}\text{4}.\text{4}\text{8}r_{\text{6}}^{-\text{3}.\text{0}\text{8}}\text{exp}\left(\frac{-\text{8}.\text{5}\text{4}}{r_{\text{6}}}\right) \end{array}$$

Elongation at rupture:


$$\begin{array}{cc} f_{\text{3}}\left(r_{i}\right)= & \text{0}.\text{3}\text{1}\text{0}\text{2}*\text{0}.\text{3}\text{7}r_{\text{1}}^{\text{0}.\text{9}\text{9}}\text{exp}\left(\frac{\text{1}\text{4}.\text{2}\text{8}}{r_{\text{1}}}\right)+\text{0}.\text{1}\text{7}\text{0}\text{3}*\text{0}.\text{3}\text{7}r_{\text{2}}^{\text{0}.\text{9}\text{9}}\text{exp}\left(\frac{\text{1}\text{4}.\text{2}\text{8}}{r_{\text{2}}}\right)+\text{0}.\text{0}\text{8}\text{9}\text{5}*\text{0}.\text{3}\text{7}r_{\text{3}}^{\text{0}.\text{9}\text{9}}\text{exp}\left(\frac{\text{1}\text{4}.\text{2}\text{8}}{r_{\text{3}}}\right)\\ & +\text{0}.\text{1}\text{7}\text{0}\text{3}*\text{0}.\text{3}\text{7}r_{\text{4}}^{\text{0}.\text{9}\text{9}}\text{exp}\left(\frac{\text{1}\text{4}.\text{2}\text{8}}{r_{\text{4}}}\right)+\text{0}.\text{1}\text{7}\text{0}\text{3}*\text{0}.\text{3}\text{7}r_{\text{5}}^{\text{0}.\text{9}\text{9}}\text{exp}\left(\frac{\text{1}\text{4}.\text{2}\text{8}}{r_{\text{5}}}\right)+\text{0}.\text{0}\text{8}\text{9}\text{5}*\text{0}.\text{3}\text{7}r_{\text{6}}^{\text{0}.\text{9}\text{9}}\text{exp}\left(\frac{\text{1}\text{4}.\text{2}\text{8}}{r_{\text{6}}}\right) \end{array}$$

It is worth noting that the infinite extension of the draft factor will cause the breakage of the primary filament or the production of hairy filament. Therefore, the key to the carbon fiber drafting process is to choose the appropriate and best drafting multiplier.

So the carbon fiber multistage drafting ratio allocation is a typical multi-objective optimization problem. And transforming the multi-objective problem into a single-objective optimization problem is the basic strategy for solving the multi-objective optimization problem. The purpose of carbon fiber drafting is to reduce the line density and increase the strength. Therefore, this paper adopts the weight summation method to form a new carbon fiber constrained objective function. The specific formula is referred to the literature [61], but due to the multi-objective transformation of single-objective intelligence to optimize the minimum value at the same time, the objective function is converted into its reciprocal, as shown in Eq. (19).


$$\text{F}=\sum_{k=\text{1}}^{\text{6}}\left(\text{1}-w)\times {(\text{log}(f}_{\text{1}}\left(r_{k}\right)/f_{\text{2}}\left(r_{k}\right))\right)+w*\text{log}\left(\frac{\text{1}}{f_{\text{3}}\left(r_{k}\right)}\right)$$ (19)

where $r_{k}$ is the six experimental parameters $r_{\text{1}}-r_{\text{6}}$. w is the weighting coefficient, which takes the value of [0,1].

### 5.3. Simulation results and analysis

According to the above description, a carbon fiber filament with good performance should have small linear density, high strength and elongation at rupture. Therefore, we propose a kernel search algorithm (LLSKSO) that optimizes the local search capability to achieve this purpose. The objective function formula is added to LLSKSO to produce the optimization results of carbon fiber parameters. The maximum number of iterations is set to be 50, the rest of the conditions are the same. The optimization results are shown in Table 8. From the table, it can be seen that the ranges of $r_{\text{1}}\sim r_{\text{6}}$ are all within the actual production range. It can be proved that the parameters optimized by LLSKSO are effective and it also shows the validity and reliability of LLSKSO.

**Table 8:** The case of LLSKSO with the value of the weight coefficient *w* in steps of 0.1.

*w*	$r_{1}$	$r_{2}$	$r_{3}$	$r_{4}$	$r_{5}$	$r_{6}$	$\rho_{L}$(tex)	$T_{g}$(CN/d)	$E_{L}$ (%)
0	4.0416	1.5086	1.4625	4.1845	1.2750	2.2655	6.4365	1.6376	9.1928
0.1	4.3110	1.9263	1.4582	5.6547	1.2619	1.2874	6.3594	1.6131	9.4871
0.2	3.8998	1.8062	1.7129	3.3707	1.2578	2.4440	6.2914	1.5943	10.4656
0.3	4.4706	1.4968	1.7574	5.3896	1.2205	1.8385	6.2747	1.5822	10.6771
0.4	4.1613	1.8649	1.2624	3.6561	1.2717	2.1478	6.0983	1.5723	10.9390
0.5	4.4992	1.8907	1.4460	5.3932	1.2988	1.5974	6.0508	1.5362	11.1743
0.6	4.0008	1.6267	1.5392	5.0469	1.2581	2.2825	6.0166	1.5132	11.2536
0.7	4.4207	1.8392	1.2464	3.3361	1.1064	1.3397	6.0064	1.4829	11.4885
0.8	4.1891	1.3508	1.1642	5.1879	1.2313	2.1077	5.9563	1.4362	11.5088
0.9	4.4599	1.8317	1.9340	3.3199	1.2819	1.5976	5.9428	1.4120	11.5262
1	3.8343	1.9899	1.8623	5.3056	1.2643	2.4149	5.9279	1.0704	13.1251

**Fig 7:**
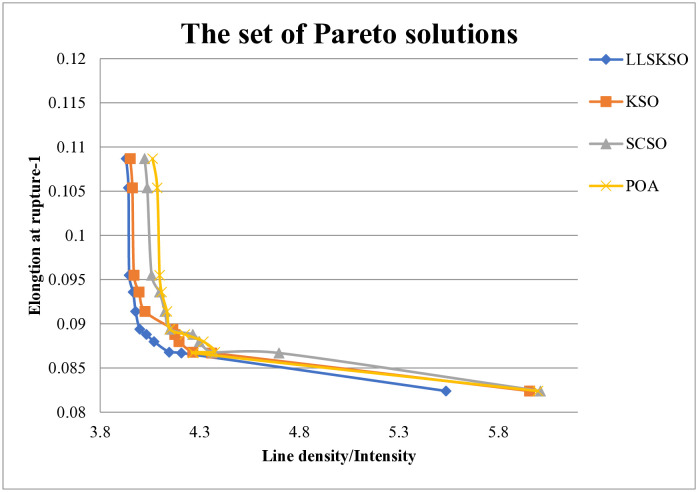
The set of Pareto solutions.

As can be seen in Table 8, when *w* = 1, it means that the line density and intensity is not considered at this time, and only the elongation at rupture is represented. When *w* = 0, it means that only the line density and intensity is represented at this time. And all three are dynamically balanced under different weighting factors. In order to fully verify the optimization performance, we draw the Pareto solution set of LLSKSO in Fig 7 against 3 compared algorithms who performed better in the benchmark function test. From Fig 7, we can see that the Pareto solution sets of all the other compared algorithms lie above LLSKSO, which illustrated that LLSKSO had better performance.

From Table 9, we can see that LLSKSO obtained the smallest value of $\frac{\rho_{L}}{T_{g}}$, 3.8905, with the smallest line density 6.4365 and the largest intensity 1.6376 at the same time. The best solutions of the compared algorithms were much larger than that of LLSKSO, and the second smallest was DBO with 4.3568, but also 11.99% larger than that of LLSKSO. For the line density $\rho_{L}$, DBO had the second smallest value of 6.7692 and 5.17% larger than LLSKSO. For the intensity, STOA had the second largest value of 1.5823 and 3.38% smaller than LLSKSO. Furthermore, LLSKSO consumed the least time among all the algorithms. It can be concluded that LLSKSO outperformed other algorithms in the comparison.

**Table 9:** Distribution scheme of draft ratio for different algorithms. (*w* = 0).

Algorithm	$r_{1}$	$r_{2}$	$r_{3}$	$r_{4}$	$r_{5}$	$r_{6}$	$\rho_{L}$(tex)	$T_{g}$(CN/d)	$\frac{\rho_{L}}{T_{g}}$	$E_{L}$(%)	Times (s)
LLSKSO	4.0416	1.5086	1.4625	4.1845	1.2750	2.2655	**6.4365**	**1.6376**	**3.8905**	9.1928	**9.1157**
KSO	4.3524	1.5092	1.6856	5.8208	1.2478	1.9957	7.4254	1.5812	4.6960	9.5834	9.1986
AOA	4.0618	1.7320	1.9845	5.7212	1.2738	2.2886	7.5920	1.5681	4.8415	9.4457	17.4568
STOA	3.6587	1.6666	1.9100	5.7073	1.2049	1.6344	7.5820	1.5823	4.7917	9.6655	15.0985
RUN	4.2243	1.1566	1.8911	3.9141	1.2876	1.3826	7.1926	1.5767	4.5618	9.3235	20.5694
CBOA	3.7598	1.4820	1.5774	5.2018	1.0107	2.4447	7.0146	1.5648	4.4827	9.2258	25.9856
DBO	2.6485	1.6068	1.2215	2.9337	1.0740	2.1217	6.7692	1.5537	4.3568	9.3606	15.2460
POA	4.3490	1.4346	1.5731	4.0917	1.2534	1.9608	7.5613	1.5749	4.8011	9.3632	10.4498
SCSO	4.4314	1.3185	1.8979	5.9612	1.2530	2.2045	6.9717	1.5694	4.4422	9.4257	10.1455

From Table 10, it can be seen that LLSKSO obtains the largest value of elongation at break of 13.1251. The comparative algorithms all have smaller elongation at break than LLSKSO. The second largest is POA with 12.3899, but it is 9.43% smaller than LLSKSO. This is followed by CBOA, SCSO etc. which are all smaller than LLSKSO. Furthermore, LLSKSO has the least time of all the algorithms with the duration of 9.0257 seconds. Therefore, all of above can prove that the results of LLSKSO have an advantage over other compared algorithms.

**Table 10:** Distribution scheme of draft ratio for different algorithms. (*w* = 1).

Algorithm	$r_{1}$	$r_{2}$	$r_{3}$	$r_{4}$	$r_{5}$	$r_{6}$	$\rho_{L}$(tex)	$T_{g}$(CN/d)	$\frac{\rho_{L}}{T_{g}}$	$E_{L}$(%)	Times(s)
LLSKSO	3.8343	1.9899	1.8623	5.3056	1.2643	2.4149	5.9279	1.0704	5.5380	**13.1251**	**9.0257**
KSO	3.8081	1.5742	1.6883	4.8081	1.2255	2.0192	5.9008	0.9988	5.9078	12.2541	9.1286
AOA	4.5000	1.5968	1.6268	5.9851	1.2898	2.0396	5.9140	0.9145	6.4669	12.3364	15.4528
STOA	4.2355	1.8746	1.7356	3.7823	1.2549	1.7318	5.9256	0.9416	6.2913	12.3656	13.0285
RUN	3.9214	1.7756	1.3494	4.3223	1.2894	1.5702	5.9365	0.9414	6.3060	12.3252	20.0984
CBOA	4.3053	1.9199	1.9596	5.8729	1.2938	2.2755	5.9998	0.9337	6.5258	12.3658	13.0126
DBO	3.4569	1.3136	1.4217	5.7628	1.2776	1.6158	5.9023	0.9611	6.1411	12.3455	13.2420
POA	3.4950	1.0678	1.5866	2.7892	1.2078	2.3804	5.9224	0.9361	6.3266	12.3899	10.4475
SCSO	3.8354	1.7081	1.4063	5.9528	1.2706	1.5376	5.9021	0.9089	6.4936	12.3581	9.9455

In summary, LLSKSO is able to obtain the best line density, strength and elongation at break compared to other algorithms under the same conditions. Although the running time of LLSKSO is not much different from that of KSO, it is lower than that of KSO in general. It is worth noting that LLSKSO and KSO are much better than RUN and AOA on the running time. So it can be proved that LLSKSO has a strong competitiveness in real engineering problems.

## 6. Conclusion

This paper proposed a novel enhancement to Kernel Search Optimization via a biologically inspired hybrid mechanism. To address the common weakness of insufficient local search precision in standard KSO models, the proposed method improves local search ability while preserving global balance. Specifically, we construct a Large Local Search KSO (LLSKSO) framework by integrating three complementary strategies: the good point set initialization, the little dung beetle search mechanism, and the Cauchy-Gauss mutation operator. Each of these is designed to boost the algorithm’s capacity to maintain diversity, explore optimal regions, and escape local traps effectively.

Compared with 10 state-of-the-art algorithms across 50 benchmark functions from the CEC suite, LLSKSO exhibits better robustness, convergence, and solution quality. For example, it achieves best-known values in 90% of the F1–F20 benchmark set, and its average CPU runtime is reduced by more than 30% compared to baseline methods. These results are supported by statistical metrics (mean, variance), visual convergence curves, and Wilcoxon Signed-Rank Test evaluations.

The MAs aim to achieve a balance between exploration and exploitation. Exploration corresponds to global search involving randomized strategies, while exploitation refers to local refinement. Increased randomness may weaken search directionality, while excessive centralization risks premature convergence. LLSKSO introduces mechanisms to address this imbalance: the good point set disperses the initial population evenly, improving the traversal capability; the little dung beetle mechanism promotes adaptive regional search inspired by biological behaviors; and the Cauchy-Gauss hybrid mutation enhances both jump strength and local fine-tuning, preserving search diversity and helping escape local optima.

To further validate practical applicability, LLSKSO was applied to the carbon fiber drafting ratio problem. The optimized parameters yielded superior performance compared to existing methods, reducing line density to 5.93 tex while enhancing tensile strength and elongation at break—demonstrating the algorithm’s capability in real-world manufacturing contexts. Moreover, LLSKSO maintains industrial usability thanks to its lightweight structure: unlike most metaheuristics that require frequent parameter calibration, LLSKSO only depends on a preset population size, making it robust and deployment-friendly.

In conclusion, LLSKSO is a competitive and reliable algorithm with strong theoretical foundations, minimal tuning complexity, and high adaptability. The experimental results on benchmark functions and industrial case studies confirm its optimization effectiveness.

In future research, LLSKSO can be studied in the following ways:

1. Continue to improve its design theory and technology based on the existing kernel mechanism (improvement mechanism). For example, more dynamically adjusting the balance between algorithm development and exploration to make it more intelligent and generalized.
2. Design a new heuristic algorithm by combining the existing improvement mechanism. And compare it with the MAs proposed in recent years to analyze its advantages, disadvantages, and performance.
3. Apply LLSKSO to machine learning, deep learning, natural language processing, and bring LLSKSO closer to the current research hotspots.

## Supporting information

S1 Data S1 Raw images. (ZIP)

## References

[1] Hui W, Feng Q. A survey of swarm intelligence optimization algorithm. Control Instrumen Chem Indust. 2007.

[2] Mavrovouniotis M, Li C, Yang S. A survey of swarm intelligence for dynamic optimization: Algorithms and applications. Swarm Evolution Comput. 2017;33:1–17. doi: 10.1016/j.swevo.2016.12.005

[3] Chen H, Yang C, Heidari AA, Zhao X. An efficient double adaptive random spare reinforced whale optimization algorithm. Exp Syst Appl. 2020;154:113018. doi: 10.1016/j.eswa.2019.113018

[4] Chen H, Zhang Q, Luo J, Xu Y, Zhang X. An enhanced Bacterial Foraging Optimization and its application for training kernel extreme learning machine. Appl Soft Comput. 2020;86:105884. doi: 10.1016/j.asoc.2019.105884

[5] Luo J, Chen H, zhang Q, Xu Y, Huang H, Zhao X. An improved grasshopper optimization algorithm with application to financial stress prediction. Appl Mathemat Modell. 2018;64:654–68. doi: 10.1016/j.apm.2018.07.044

[6] Chen H, Xu Y, Wang M, Zhao X. A balanced whale optimization algorithm for constrained engineering design problems. Appl Mathemat Modell. 2019;71:45–59. doi: 10.1016/j.apm.2019.02.004

[7] Luo J, Chen H, Heidari AA, Xu Y, Zhang Q, Li C. Multi-strategy boosted mutative whale-inspired optimization approaches. Appl Mathemat Modell. 2019;73:109–23. doi: 10.1016/j.apm.2019.03.046

[8] Chen H, Wang M, Zhao X. A multi-strategy enhanced sine cosine algorithm for global optimization and constrained practical engineering problems. Appl Mathemat Comput. 2020;369:124872. doi: 10.1016/j.amc.2019.124872

[9] Zhang X, Xu Y, Yu C, Heidari AA, Li S, Chen H, et al. Gaussian mutational chaotic fruit fly-built optimization and feature selection. Exp Syst Appl. 2020;141:112976. doi: 10.1016/j.eswa.2019.112976

[10] Blum C. Ant colony optimization: Introduction and recent trends. Phys Life Rev. 2005;2(4):353–73. doi: 10.1016/j.plrev.2005.10.001

[11] Clerc M. Particle Swarm Optimization. 2006.

[12] Yang XS, He X. Firefly algorithm: recent advances and applications. IJSI. 2013;1(1):36. doi: 10.1504/ijsi.2013.055801

[13] Dhiman G, Kumar V. Seagull optimization algorithm: Theory and its applications for large-scale industrial engineering problems. Knowl-Based Syst. 2019;165:169–96. doi: 10.1016/j.knosys.2018.11.024

[14] Mirjalili S, Lewis A. The whale optimization algorithm. Adv Eng Soft. 2016;95:51–67.

[15] Heidari AA, Mirjalili S, Faris H, Aljarah I, Mafarja M, Chen H. Harris hawks optimization: Algorithm and applications. Future Generat Comput Syst. 2019;97:849–72. doi: 10.1016/j.future.2019.02.028

[16] Song W. et al. An Improved Sparrow Search Algorithm. In: 2020 IEEE Intl Conf on Parallel & Distributed Processing with Applications, Big Data & Cloud Computing, Sustainable Computing & Communications, Social Computing & Networking (ISPA/BDCloud/SocialCom/SustainCom). 2020.

[17] Li WK, Wang WL, Li L. Optimization of Water Resources Utilization by Multi-Objective Moth-Flame Algorithm. Water Resour Manage. 2018;32(10):3303–16. doi: 10.1007/s11269-018-1992-7

[18] Sapre S, Mini S. Opposition-based moth flame optimization with Cauchy mutation and evolutionary boundary constraint handling for global optimization. Soft Comput. 2018;23(15):6023–41. doi: 10.1007/s00500-018-3586-y

[19] Xu Y, Chen H, Heidari AA, Luo J, Zhang Q, Zhao X, et al. An efficient chaotic mutative moth-flame-inspired optimizer for global optimization tasks. Exp Syst Appl. 2019;129:135–55. doi: 10.1016/j.eswa.2019.03.043

[20] Wang M, Chen H, Yang B, Zhao X, Hu L, Cai Z, et al. Toward an optimal kernel extreme learning machine using a chaotic moth-flame optimization strategy with applications in medical diagnoses. Neurocomputing. 2017;267:69–84. doi: 10.1016/j.neucom.2017.04.060

[21] Adarsh BR, Raghunathan T, Jayabarathi T, Yang X-S. Economic dispatch using chaotic bat algorithm. Energy. 2016;96:666–75. doi: 10.1016/j.energy.2015.12.096

[22] Liu W, et al. A novel sigmoid-function-based adaptive weighted particle swarm optimizer. IEEE Transact Cybernet. 2021;51(2):1085–93. 10.1109/TCYB.2019.2925015 31329142

[23] Liu W, Wang Z, Zeng N, Yuan Y, Alsaadi FE, Liu X. A novel randomised particle swarm optimizer. Int J Mach Learn Cyber. 2020;12(2):529–40. doi: 10.1007/s13042-020-01186-4

[24] Jia H. An improved particle swarm algorithm incorporating multiple strategies. Comput Syst Appl. 2021;30(7):6.

[25] Tian L, Dexin C. Improved simplified particle swarm algorithm based on levy flight. Comput Eng Appl. 2021;57(20):188–96.

[26] Rui W, Jinguo W, Na W. Research on path planning of mobile robot based on improved ant colony algorithm. In: Joint International Mechanical, Electronic and Information Technology Conference. 2015.

[27] Liu Y, Cao B, Li H. Improving ant colony optimization algorithm with epsilon greedy and Levy flight. Complex Intell Syst. 2020;7(4):1711–22. doi: 10.1007/s40747-020-00138-3

[28] Xiong C, Yi Z, Jianda H. An improved ant colony algorithm for robot path planning. Control Theory Appl. 2010;27(6):5.

[29] Beichen Y, aftermath. Application of improved ant colony algorithm in path planning. Comput Appl Res. 2022;39(11):3292–7.

[30] Tao R, Meng Z, Zhou H. A self-adaptive strategy based firefly algorithm for constrained engineering design problems. Appl Soft Comput. 2021;107:107417. doi: 10.1016/j.asoc.2021.107417

[31] Yelghi A, Köse C. A modified firefly algorithm for global minimum optimization. Appl Soft Comput. 2018;62:29–44. doi: 10.1016/j.asoc.2017.10.032

[32] Wang C-F, Song W-X. A novel firefly algorithm based on gender difference and its convergence. Appl Soft Comput. 2019;80:107–24. doi: 10.1016/j.asoc.2019.03.010

[33] Hussain K, Zhu W, Mohd Salleh MN. Long-Term Memory Harris’ Hawk Optimization for High Dimensional and Optimal Power Flow Problems. IEEE Access. 2019;7:147596–616. doi: 10.1109/access.2019.2946664

[34] Dexin Y, et al. Harris hawk algorithm based on chaotic lens imaging learning and its application. J Sens Technol. 2021;34(11):12.

[35] Cheng Z, Xuhua P, Yong Z. Harris hawk optimization algorithm based on convergence correction. Comput Appl. 2022;42(4):1186–93.

[36] Xiaolong L, Tongying L. Harris hawk optimization algorithm based on square neighborhoods and random arrays. Control Decis Mak. 2022;37(10):10.

[37] Chengtian O, Yujia L, Donglin Z. An adaptive chaotic sparrow search optimization algorithm. In: 2021 IEEE 2nd International Conference on Big Data, Artificial Intelligence and Internet of Things Engineering (ICBAIE). 2021.

[38] Gao S, Wang X, Jin C. Analysis of Multi-Threshold Image Segmentation Method Based on Improved Sparrow Search Algorithm. In: 2022 4th International Conference on Applied Machine Learning (ICAML). 2022.

[39] Jie Y, Tuo F, Yaoping Z. UAV Track Planning Based on Improved Sparrow Search Algorithm. In: 2022 4th International Conference on Natural Language Processing (ICNLP). 2022.

[40] Ouyang C, Zhu D, Wang F. A learning sparrow search algorithm. Computat Intell Neurosci. 2021;3946958. 10.1155/2021/3946958 PMC8369163 34413887

[41] Wolpert DH, Macready WG. No free lunch theorems for optimization. IEEE Trans Evol Computat. 1997;1(1):67–82. doi: 10.1109/4235.585893

[42] Dong R, Ma L, Chen H, Heidari AA, Liang G. Hybrid kernel search and particle swarm optimization with Cauchy perturbation for economic emission load dispatch with valve point effect. Front Energy Res. 2023;10. doi: 10.3389/fenrg.2022.1061408

[43] Dong R, Wang S. New Optimization Algorithm Inspired by Kernel Tricks for the Economic Emission Dispatch Problem With Valve Point. IEEE Access. 2020;8:16584–94. doi: 10.1109/access.2020.2965725

[44] Dong R, Chen H, Heidari AA, Turabieh H, Mafarja M, Wang S. Boosted kernel search: Framework, analysis and case studies on the economic emission dispatch problem. Knowl-Based Syst. 2021;233:107529. doi: 10.1016/j.knosys.2021.107529

[45] Kazimipour B, Li X, Qin AK. A review of population initialization techniques for evolutionary algorithms. In: 2014 IEEE Congress on Evolutionary Computation (CEC). 2014.

[46] Zhu F, Li G, Tang H, Li Y, Lv X, Wang X. Dung beetle optimization algorithm based on quantum computing and multi-strategy fusion for solving engineering problems. Exp Syst Appl. 2024;236:121219. doi: 10.1016/j.eswa.2023.121219

[47] Jung M. A mutational image denoising model under mixed Cauchy and Gaussian noise. AIMS Mathematics. 2022;7:19696-19726.

[48] Ahmadianfar I, Heidari AA, Gandomi AH, Chu X, Chen H. RUN beyond the metaphor: An efficient optimization algorithm based on Runge Kutta method. Exp Syst Appl. 2021;181:115079. doi: 10.1016/j.eswa.2021.115079

[49] Dhiman G, Kaur A. STOA: A bio-inspired based optimization algorithm for industrial engineering problems. Eng Appl Artific Intell. 2019;82:148–74. doi: 10.1016/j.engappai.2019.03.021

[50] Zhang X, Feng T. Chaotic bean optimization algorithm. Soft Comput. 2018;22(1):67–77. doi: 10.1007/s00500-016-2322-8

[51] Chaudhary R, Banati H. Peacock Algorithm. 2019.

[52] Abdel-Basset M, Mohamed R, Jameel M, Abouhawwash M. Nutcracker optimizer: A novel nature-inspired metaheuristic algorithm for global optimization and engineering design problems. Knowl-Based Syst. 2023;262:110248. doi: 10.1016/j.knosys.2022.110248

[53] Abualigah L, et al. The Arithmetic Optimization Algorithm. Comput Method Appl Mechan Eng. 2021;376:113609.

[54] Dehghani M, Montazeri Z, Trojovská E, Trojovský P. Coati Optimization Algorithm: A new bio-inspired metaheuristic algorithm for solving optimization problems. Knowl-Based Syst. 2023;259:110011. doi: 10.1016/j.knosys.2022.110011

[55] Seyyedabbasi A, Kiani F. Sand Cat swarm optimization: a nature-inspired algorithm to solve global optimization problems. Eng Comput. 2022;39(4):2627–51. doi: 10.1007/s00366-022-01604-x

[56] Dong R, Wang S. New optimization algorithm inspired by fluid mechanics for combined economic and emission dispatch problem. Turk J Elec Eng Comp Sci. 2018;26(6):3306–19. doi: 10.3906/elk-1803-88

[57] Jiajia C. Collaborative modeling and intelligent optimization of carbon fiber spinning process. Donghua University; 2013.

[58] Radishevskii MB, Serkov AT. Coagulation Mechanism in Wet Spinning of Fibres. Fibre Chem. 2005;37(4):266–71.

[59] Jiajia C. Effects of stretching on the structure and properties of polyacrylonitrile primary filaments. Synthetic Fiber. 2002;31(5):3.

[60] Boor D. A practical guide to splines. 1978.

[61] Jiajia C, et al. A multi-objective dynamic programming approach for carbon fiber stretching process optimization. Materials Herald. 2011;25(6):4.

